# ATP-binding cassette transporters mediate differential biosynthesis of glycosphingolipid species

**DOI:** 10.1016/j.jlr.2021.100128

**Published:** 2021-09-28

**Authors:** Monique Budani, Christiane Auray-Blais, Clifford Lingwood

**Affiliations:** 1 Division of Molecular Medicine, Research Institute, Hospital for Sick Children, Toronto, Ontario, Canada; 2 Department of Laboratory Medicine & Pathobiology, University of Toronto, Toronto, Ontario, Canada; 3 Division of Medical Genetics, Department of Pediatrics, Faculty of Medicine and Health Sciences, Université de Sherbrooke, Québec, Canada; 4 Department of Biochemistry, University of Toronto, Toronto, Ontario, Canada

**Keywords:** ABC transporter, glucosylceramide flippase, glycosphingolipid, photoprobes, metabolic labeling, LacCer, GlcCer pools, metabolic channeling, GSL anabolism, GlcCer synthase, B3GALT1, β-1,3-galactosyltransferase 1, B4GALT6, β-1,4-galactosyltransferase 6, Cer, ceramide, FAPP2, phosphatidylinositol-four-phosphate adapter protein 2, GCS, GlcCer synthase, GlcCer, glucosylceramide, GLTP, glycolipid transfer protein, GSL, glycosphingolipid, KD, knockdown, LacCer, lactosylceramide, Lc_3_, lactotriaosylceramide, LCS, LacCer synthase, NBD, nitrobenzo-2-oxa-1,3-diazole, NC, negative control, siRNA, small interfering RNA, ST3GAL5, β-galactoside α-2,3-sialyltransferase 5, GM3 synthase, TMD, transmembrane domain

## Abstract

The cytosolic-oriented glucosylceramide (GlcCer) synthase is enigmatic, requiring nascent GlcCer translocation to the luminal Golgi membrane to access glycosphingolipid (GSL) anabolic glycosyltransferases. The mechanism by which GlcCer is flipped remains unclear. To investigate the role of GlcCer-binding partners in this process, we previously made cleavable, biotinylated, photoreactive GlcCer analogs in which the reactive nitrene was closely apposed to the GlcCer head group, while maintaining a C16-acyl chain. GlcCer-binding protein specificity was validated for both photoprobes. Using one probe, XLB, here we identified ATP-binding cassette (ABC) transporters ABCA3, ABCB4, and ABCB10 as unfractionated microsomal GlcCer-binding proteins in DU-145 prostate tumor cells. siRNA knockdown (KD) of these transporters differentially blocked GSL synthesis assessed in toto and via metabolic labeling. KD of ABCA3 reduced acid/neutral GSL levels, but increased those of LacCer, while KD of ABCB4 preferentially reduced neutral GSL levels, and KD of ABCB10 reduced levels of both neutral and acidic GSLs. Depletion of ABCA12, implicated in GlcCer transport, preferentially decreased neutral GSL levels, while ABCB1 KD preferentially reduced gangliosides, but increased neutral GSL Gb_3_. These results imply that multiple ABC transporters may provide distinct but overlapping GlcCer and LacCer pools within the Golgi lumen for anabolism of different GSL series by metabolic channeling. Differential ABC family member usage may fine-tune GSL biosynthesis depending on cell/tissue type. We conclude that ABC transporters provide a new tool for the regulation of GSL biosynthesis and serve as potential targets to reduce selected GSL species/subsets in diseases in which GSLs are dysregulated.

Glycosphingolipids (GSLs) have many functional, structural, and regulatory roles (1). They are involved in cell signaling, apoptosis, cell differentiation, proliferation, cell adhesion, and pathogen entry (1, 2). Aberrant GSL metabolism is pathologically associated with many diseases such as GSL storage diseases (3), type II diabetes (4), atherosclerosis (5), lupus (6), asthma (7), cancer (8, 9, 10), multiple drug-resistant tumors (11, 12), metabolic syndrome (13), inflammation (14), and the neuropathies, ALS (15), Alzheimer's and Parkinson's disease (16, 17). Inhibition of GSL synthesis alleviates Gaucher disease symptoms (18) and prevents or reverses the phenotype in models of diseases associated with GSLs (7, 19, 20, 21). In addition, GSL biosynthesis is required for SARS-CoV-2 and influenza viral propagation (22). Understanding complex GSL synthesis is therefore crucial in generating the means for selective therapeutic correction of GSL levels. Currently, the only approach is to inhibit all GSLs using glucosylceramide synthase (GCS) inhibitors such as Miglustat (23), and eliglustat (24, 25), which can cause adverse side effects (26, 27, 28, 29).

Glucosylceramide (GlcCer) is the precursor of >90% of mammalian GSLs, while galactosylceramide and fucosylceramide account for the remainder (1). GlcCer is synthesized from ceramide (Cer) by GCS on the cytosolic leaflet of the Golgi (30, 31, 32). GCS activity is widely distributed throughout the Golgi, with highest specific activity in the cis/medial-Golgi (31). Lactosylceramide (LacCer) is generated from GlcCer by LacCer synthase (LCS) on the Golgi luminal leaflet (33, 34). LCS is localized throughout the Golgi (34, 35) and TGN (36). LacCer is the diverging point for conversion into more complex GSLs - ganglio, globo, lacto, and neolacto series (1), also made within the Golgi lumen (34). However, the mechanism by which GlcCer translocates into the Golgi lumen still remains largely a matter of conjecture (36, 37).

A few GlcCer transport pathways have been proposed thus far. In nonvesicular GlcCer traffic, phosphatidylinositol-four-phosphate adapter protein 2 (FAPP2) transports cytosolic GlcCer from the *cis*-Golgi to the TGN, where it is translocated by a proposed ATP-dependent GlcCer flippase (36), possibly multidrug resistance protein 1 (MDR1, Pgp, ABCB1), to the luminal leaflet for neutral (i.e., globo-series) GSL synthesis (36). For acidic (i.e., gangliosides) GSL synthesis, GlcCer synthesized on the cytosolic leaflet of early Golgi membranes is translocated to the lumen by an uncharacterized ATP-dependent flippase and delivered to the TGN via vesicular transport (2, 36). Alternatively, cytosolic GlcCer may undergo retrograde transport back to the ER via FAPP2, flipped by an ATP-independent flippase to the ER lumen, and transported to the TGN by vesicular transport (35). However, this has since been contradicted by studies showing that FAPP2 knockdown does not decrease GM_3_ synthesis (36).

ABCB1 is an ATP-dependent drug efflux pump, which facilitates drug resistance in tumor cells (38). It is a member of the ATP-binding cassette (ABC) transporter superfamily, containing two cytoplasmic nucleotide-binding domains and two transmembrane domains (TMDs) (39). ABCB1 was predicted to have more than 300 substrates (40), with a diverse range including drugs, peptides, phospholipids, and sphingolipids (41, 42). ABCB1-transfected epithelial LLC-PK1 cells translocated C6-NBD (nitrobenzo-2-oxa-1,3-diazole)-GlcCer, C6-GlcCer, and C8C8-GlcCer across the apical membrane, which was reduced by MDR inhibitors and energy depletion (43). However, ABCB1 has been found not only at the plasma membrane but also the Golgi complex (44). ABCB1 was first suggested as a potential mechanism for flipping GlcCer to the luminal leaflet of the Golgi for GSL biosynthesis when GSL levels were increased in ABCB1 retroviral cell transfection (45). Further studies have shown that ABCB1 translocates short-chained fluorescent GlcCer when reconstituted in proteoliposomes (42), which was inhibited by addition of C12-GlcCer (46). The first evidence of differential GSL anabolic regulation was our inhibition of LacCer and Gb_3_, but not ganglioside, biosynthesis in ABCB1-expressing cell lines by cyclosporin A (47), supporting an ABCB1 role as a flippase selectively involved in globo-series GSL biosynthesis. However, cyclosporin A is not a specific ABCB1 inhibitor. The role of ABCB1 as a GlcCer flippase has been challenged, suggesting that it only translocates short-chain GlcCer and not natural GlcCer (35). Interestingly, GCS overexpression in cancer has been shown to correlate with ABCB1 expression (48), and increased ABCB1 correlates with increased complex GSLs in drug-resistant cells (49). Inhibiting GCS activity blocks ABCB1 overexpression (50), and vice versa (51), resensitizing drug-resistant cells to chemotherapeutic drugs. This can involve additional ABC transporters (52). The mechanism by which ABCB1 flips phospholipid/GSL substrates remains unclear (53), but was suggested to resemble its drug efflux mechanism (39, 41, 42).

The role of ABCB1 in GlcCer luminal translocation remains ill-defined and is unlikely the only mechanism. Other ABC proteins (ABCA12) and P4-ATPases (ATP10A and ATP10D) have been implicated in GlcCer translocation, but have yet to be investigated as Golgi flippases in GSL biosynthesis (54, 55, 56). ABCA12 is essential for keratinocyte differentiation and maintaining the skin lipid barrier (55). Formation of the intercellular lipid layers is vital for epidermal barrier function; therefore, barrier function is lost if lipid layer formation is defective (55). ABCA12 transports GlcCer to the inner leaflet of epidermal keratinocyte lamellar granules for secretion (57). ABCA12 deficiency decreases epidermal Cer, disrupts GlcCer lamellar granule accumulation, and increases gangliosides in keratinocytes (57). It is suggested that ganglioside accumulation in keratinocytes causes apoptosis (55). Severe ABCA12 protein defects result in harlequin ichthyosis, a congenital skin disorder caused by skin lipid barrier loss (55). Immunofluorescent staining with ABCA12, GlcCer, and Golgi apparatus antibodies showed colocalization of ABCA12 and GlcCer within the granular layer of keratinocytes and showed ABCA12 distribution from the Golgi apparatus to cell periphery (58). Whether ABCA12 plays a role in GlcCer metabolism in nonepidermal tissues is unknown, but ABCA12 expression is not limited to the skin (59). Human P4-ATPases ATP10A and ATP10D were found to transport short-chain NBD-GlcCer (54). ATP10D single-nucleotide polymorphisms are associated with GlcCer elevation in plasma, and both ATP10A and ATP10D are linked to metabolic disease (60).

It is probable that other unidentified flippases are involved in Golgi GlcCer transport for GSL biosynthesis in addition to these other ill-defined GlcCer membrane transport proteins. Multiple Golgi and TGN flippases could be responsible for the formation of different GlcCer pools from which acidic and neutral or specific GSL series are derived. Defining such flippases would close a major gap in understanding GSL biosynthesis, perhaps providing new opportunities for more selective drug therapy to treat diseases affected by aberrant GSL metabolism. In this study, we used novel GlcCer-based photoaffinity probes, XLA and XLB (61), to identify GlcCer-binding proteins as candidate GlcCer flippases and investigated their roles in GSL biosynthesis.

## Materials and methods

### Reagents

2X PCR TaqFast MasterMix was purchased from Applied Biological Materials Inc. (abm). ^14^C-galactose was purchased from American Radiolabelled Chemicals. 4-(2-Aminoethyl)benzenesulfonyl fluoride hydrochloride (AEBSF), protease inhibitor cocktail (50 μM AEBSF, 40 nM aprotinin, 25 μM bestatin, 75 nM E-64, 1 μM leupeptin, 0.5 μM pepstatin A), Tris, MgCl_2_, MnCl_2_, and sucrose were purchased from BioShop. Chloroform, KCl, and methanol were purchased from Caledon Laboratory Chemicals. siRNA was purchased from GenePharma. 10X DNase I Reaction Buffer (200 mM Tris-HCl pH, 500 mM KCl, 20 mM MgCl_2_), DNase I Amp Grade 1 U/μl, 25 mM EDTA, Oligo (dT) 20, dNTP Mix (10 mM each dATP, dGTP, dCTP and dTTP at neutral pH), 5X First-Strand Buffer (250 mM Tris-HCl pH 8.3, 375 mM KCl, 15 mM MgCl_2_), 0.1 M DTT, Lipofectamine® RNAiMAX Transfection Reagent, RNaseOUT Recombinant RNase Inhibitor, Streptavidin T1 Magnetic Dynabeads, and SuperScript III Reverse Transcriptase (RT) were purchased from Invitrogen. Precoated TLC sheets (Polygram SIL G/UV254) were purchased from Machery-Nagel. Glucosylceramide (glucocerebrosides) was purchased from Matreya LLC. HCl, iodoacetamide, Mg(OAc)_2_, NaOH, were purchased from Sigma-Aldrich. BCA Protein Assay Kit, Opti-MEM® I Reduced Serum Medium, sequencing grade modified trypsin porcine 20 μg, and TRIzol® Reagent were purchased from Thermo Fisher Scientific. Conduritol β epoxide (CBE) was purchased from Toronto Research Chemicals. Sep-Pak Vac 6 cc (1 g) certified C18 cartridges were purchased from Waters. Minimum Essential Medium (MEM), Phosphate-Buffered Saline (D-PBS), Fetal Bovine Serum (FBS), Trypsin (0.05%)/EDTA were purchased from Wisent Inc. DU-145 cells were kindly supplied by Dr N. Fleshner, University of Toronto. GLTP was kindly provided by Dr Thorsten Lang, Department of Membrane Biochemistry at the Life & Medical Sciences (LIMES) Institute, University of Bonn, Germany.

### Cell culture

DU-145 prostate cancer cells were grown in MEM supplemented with 10% FBS at 37°C and passaged using D-PBS and 0.05% trypsin/EDTA.

### Preparation of unfractionated microsomes

As in (61), DU-145 cells detached by 0.05% trypsin/EDTA were collected in equal volume of FBS to inhibit trypsin. Cells were washed twice with ice-cold D-PBS (360 *g*, 4°C), and cell pellets were stored at –80°C. DU-145 pellets were suspended in ice-cold homogenization buffer (10 mM Tris-HCl pH 7.4, 10 mM KCl, 1.5 mM MgCl_2_, 0.5 M sucrose), and homogenized with 30 strokes of a Dounce homogenizer. The homogenized sample was centrifuged (1,000 *g* for 10 min at 4°C) to pellet nuclei and debris. The supernatant was centrifuged at 10,000 *g* for 10 min at 4°C. The protein concentration of the collected supernatant (crude microsomes) was determined with BCA protein assay and stored at –80°C as 200 μg aliquots with 0.1 mM AEBSF (protease inhibitor). This unfractionated microsome preparation method is a modification of De Rosa *et al.* (47) to retain a mixture of Golgi, ER, plasma membrane, and cytosol, so all necessary components for GSL biosynthesis would be present. DTT was also omitted to avoid reduction of the cleavable disulfide bond in XLA and XLB.

### Unfractionated microsomal protein cross-linking assay

To identify putative GlcCer flippases, XLA and XLB were delivered by GLTP and cross-linked to DU-145 unfractionated microsomal proteins as in (61). In dark conditions, 0.1 μg XLA or XLB was dried and suspended in water via sonication and stored at –20°C until use. Control (no GlcCer analog cross-linker), XLA, and XLB samples were preincubated with 2 mM GLTP or water of equal volume at 37°C for 1 h. To mimic a LacCer synthase assay in detergent-free conditions, samples were incubated with or without 100 μg DU-145 unfractionated microsomes, and 1 mM MnCl_2_, 1 mM Mg(OAc)_2_, 20 mM cacodylate pH 6.8, protease inhibitor cocktail (50 μM AEBSF, 40 nM aprotinin, 25 μM bestatin, 75 nM E-64, 1 μM leupeptin, 0.5 μM pepstatin A), 0.5 mM UDP-Gal, and 0.25 mM CBE at 37°C for 1 h. Proteins were cross-linked with Spectroline Model EB-280C UV (302 nm) from a distance of 5 cm for 15 min. Samples were prepared for proteomic analysis.

### Proteomics

To identify putative GlcCer flippases, XLA and XLB were delivered by GLTP and cross-linked to DU-145 unfractionated microsomal proteins as in (61). Samples were solubilized with 1X radioimmunoprecipitation assay (RIPA) buffer (1X TBS, 1 mM EDTA, 1 mM EGTA, 0.1% SDS, 1% Triton X-100, 0.5% sodium deoxycholate) and centrifuged at 10,000 *g* for 10 min at room temperature. Supernatant containing cross-linked biotinylated proteins was collected and purified with MyOne Streptavidin T1 Magnetic Dynabeads. Before purification, Dynabeads were vortexed for 30 s, aliquoted into 1.5 ml microtubes, washed with 1X RIPA buffer three times using μMACS Separation Unit magnet. After Dynabeads were washed, they were incubated with cross-linked protein samples with shaking for 30 min at room temperature. Unbound supernatant was decanted, and beads were washed with 1X RIPA buffer three times and D-PBS two times. Protein-coupled beads were stored at –20°C until use. Protein-bound bead samples were denatured in 8 M urea, 50 mM Tris–HCl pH 8, and 4 mM DTT, heated at 60°C for 45 min. Samples were cooled to room temperature, and cysteine residues were alkylated with 10 mM iodoacetamide incubated in the dark at room temperature for 15 min. Samples were diluted with 50 mM NH_4_HCO_3_ pH 7.8 until urea concentration was less than 1 M. CaCl_2_ from 100 mM stock was added to make a final concentration of 1 mM. Sequencing grade modified porcine trypsin was dissolved in 100 μl 0.01% TFA (0.2 μg/μl), aliquoted into 10 μl shots, and stored at –20°C until use. Proteins were digested by 1:100 (w/w) trypsin:protein, incubated at 37°C with shaking for 24 h. Samples were analyzed by LC/MS/MS Orbitrap-Elite, performed by the SPARC BioCentre (Molecular Analysis), The Hospital for Sick Children, Toronto, Canada.

### Experimental design and statistical rationale

For identification of potential GlcCer flippases involved in GSL biosynthesis, GlcCer analog photoprobe stereoisomers XLA and XLB were cross-linked in DU-145 cell unfractionated microsomes. This human prostate carcinoma cell line was used because of its extensive acidic and neutral GSL content. The sample size was n = 1, and there were no replicates performed, which was acceptable for our study as the GlcCer cross-linker probes (XLA and XLB) were only used to identify GlcCer-binding proteins as candidate GlcCer flippases. Actual candidate flippase involvement in GSL biosynthesis would be confirmed by cellular knockdown studies.

There were three different controls employed. “Control” sample with unfractionated microsomes but without cross-linker to rule out endogenous biotinylated proteins. “XLA + GLTP” and “XLB + GLTP” samples, contained as stated, but without unfractionated microsomes. These were used to exclude any contaminated proteins present in GLTP when GLTP was used for cross-linker delivery and insertion into the microsomal membranes.

### Tandem mass spectrometry search parameters and acceptance criteria

Peaks were assigned via Proteome Discoverer version 1.4.0.288. Charge state deconvolution and deisotoping were not performed. All MS/MS samples were analyzed using Sequest (Thermo Fisher Scientific, San Jose, CA; version 1.4.0.288) and X! Tandem (The GPM, thegpm.org; version CYCLONE (2010.12.01.1)). Sequest was set up to search Uniprot-Human-Nov12015.fasta (unknown version, 42,087 entries) assuming the digestion enzyme trypsin with 2 max missed cleavages. X! Tandem was set up to search a subset of the Uniprot-Human-Nov12015 database (unknown version, 84,264 entries) also assuming trypsin with 2 max missed cleavages. Sequest and X! Tandem were searched with a fragment ion mass tolerance of 0.60 Da and a parent ion tolerance of 10.0 PPM. Carbamidomethyl of cysteine was specified in Sequest and X! Tandem as a fixed modification. Deamidated of asparagine and glutamine and oxidation of methionine were specified in Sequest as variable modifications. Glu->pyro-Glu of the N-terminus, ammonia-loss of the N-terminus, gln->pyro-Glu of the N-terminus, deamidated of asparagine and glutamine, and oxidation of methionine were specified in X! Tandem as variable modifications.

Scaffold (version Scaffold_4.8.7, Proteome Software Inc., Portland, OR) was used to validate MS/MS-based peptide and protein identifications. Peptide identifications were accepted if they could be established at greater than 95.0% probability by the Scaffold Local FDR algorithm. Protein identifications were accepted if they could be established at greater than 95.0% probability and contained at least 1 identified peptide. Protein probabilities were assigned by the Protein Prophet algorithm (62). Proteins that contained similar peptides and could not be differentiated based on MS/MS analysis alone were grouped to satisfy the principles of parsimony.

### siRNA transfection

DU-145 cells were transfected in 6-well plates at 80% confluency. The highest of the suggested 60%–80% confluency range from the transfection reagent protocol was used to allow for continued growth while maximizing cell number by the end of transfection. Lipofectamine RNAiMAX Transfection Reagent (75 pmol) and 75 pmol siRNA (see supplemental Table S1 for sequences) were diluted in 250 μl Opti-MEM I Reduced Serum Medium and incubated at room temperature for 5 min. The siRNA-reagent complex (250 μl) was added to each well containing 2 ml of EMEM +10% FBS. Cells were incubated at 37°C for 24 h, washed with D-PBS, and incubated at 37°C in fresh EMEM + 10% FBS for 24 h. Cells were then used for subsequent GSL extraction or GSL metabolic labeling, and transfection efficacy was analyzed by RNA extraction and RT-PCR.

### Glycosphingolipid metabolic labeling

Post transfection, cells were incubated with 1.5 μCi ^14^C-galactose diluted in 2 ml/well MEM +10% FBS for 5 h at 37°C. Metabolic labeling studies of cultured cell GSLs at one relatively short time interval were used to maximize synthetic, as opposed to turnover contribution of cellular GSLs (63, 64, 65). Prior to GSL extraction, cells were washed with D-PBS twice.

### RNA extraction

For dissociation of the nucleoprotein complex, media was removed from 6-well plate, replaced with 1 ml/well of TRIzol Reagent, and incubated at room temperature for 5 min. Samples were transferred to 1.5 ml microtubes and mixed vigorously by hand for 15 s with 0.2 ml chloroform. Samples were incubated at room temperature for 3 min, centrifuged at 12,000 *g* for 15 min at 4°C, and the aqueous phase containing RNA was transferred to a new 1.5 ml microtube. To precipitate the RNA, samples were incubated at room temperature with 0.5 ml isopropanol for 10 min. RNA was collected by centrifugation at 12,000 *g* for 10 min, and supernatant was discarded. RNA pellet was washed in 1 ml of 75% ethanol and centrifuged at 7,500 *g* for 5 min at 4°C. After supernatant was discarded, RNA pellet was vacuum dried. RNA was suspended in DEPC-treated water and incubated at 60°C for 10 min. The RNA was quantified with NanoDrop 2000c Spectrophotometer and stored at –80°C until use.

### DNase digestion

For DNA digestion of single and double-stranded DNA, 1 μg RNA sample was incubated at room temperature for 10 min with 1 μl 10× DNase I Reaction Buffer, 1 μl DNase I Amp Grade 1 U/μl, and DEPC-treated water to final volume of 10 μl. DNase I was inactivated by incubation with 1 μl of 25 mM EDTA at 65°C for 10 min.

### First-strand synthesis

For first-strand synthesis of cDNA, 1 μg DNase I treated RNA samples were incubated with 1 μl oligo (dT) 20, and 1 μl dNTP Mix at 65°C for 5 min, and incubated on ice for 5 min. Samples were briefly centrifuged and incubated with 4 μl 5X First-Strand Buffer, 1 μl 0.1 M DTT, 1 μl RNaseOUT Recombinant RNase Inhibitor, and SuperScript III RT at 50°C for 60 min. Reaction was inactivated by incubation at 70°C for 15 min. Synthesized cDNA was stored at –20°C until use as a template for RT-PCR amplification.

### Reverse transcription polymerase chain reaction

RT-PCR was used for cDNA amplification. cDNA templates from first-strand synthesis (1.5 ng) were amplified using 400 nM forward and reverse primers (supplemental Table S2), 1X PCR TaqFast MasterMix with dye, and DEPC-treated water to a final volume of 50 μl. Samples were incubated in thermal cycler programmed to the following: Step 1/initial denaturation: 3 min at 94°C. Step 2/denaturation: 30 s at 94°C. Step 3/annealing: 30 s at primer specific temperature. Step 4/extension: 1 min at 72°C. Step 5: Repeat steps 2–4 for primer-specific number of cycles. Step 6/final extension: 10 min at 72°C. Step 7/final holding: 4°C until use or storage at –20°C. Amplification products were analyzed by gel electrophoresis with 1% agarose gel in 0.5X TAE and stained with GelRed (1:10,000, v/v). Gels were imaged with BioRad ChemDoc MP Imaging System (Nucleic Acid GelRed setting).

### Glycosphingolipid extraction

Post transfection, 6-well plates containing DU-145 cells in EMEM +10% FBS were washed with D-PBS and incubated in 1 ml 0.5 N NaOH/methanol at 37°C for 1 h to saponify phospholipids. Samples were transferred to glass test tube, neutralized with aqueous 0.5 N HCl, diluted with water (total volume less than 30% methanol), and desalted by C18 Sep-Paks. GSL extracts were dried under nitrogen and low heat and if necessary stored at –20°C until use.

### Thin-layer chromatography

GSLs extracted from 6-well plates were dissolved in CHCl_3_:CH_3_OH (2:1, v/v) before loading to silica-coated TLC plate with Hamilton syringe. Samples were resolved first with CHCl_3_:CH_3_OH (98:2, v/v) mobile phase, dried, and subsequently resolved with CHCl_3_:CH_3_OH:H_2_O (60:40:8, v/v/v). For total GSL analysis, samples were visualized by staining with 0.5% orcinol in 3 M H_2_SO_4_ and incubation at 130°C. Metabolically labeled GSLs were exposed to film for 2 days and developed for visualization. GSL bands were quantified using ImageJ. All knockdowns were calculated as a percentage of the mean value of NC for each trial and plotted with GraphPad Prism 7.

### Analysis of Gb_3_ isoforms

DU-145 cell Gb_3_ isoforms were analyzed post ABC transporter siRNA transfection by normal-phase ultraperformance liquid chromatography coupled to tandem mass spectrometry (UPLC-MS/MS) as described (66). Results were plotted using GraphPad Prism 7.

## Results

### Proteomic analysis

**Fig. 1:**
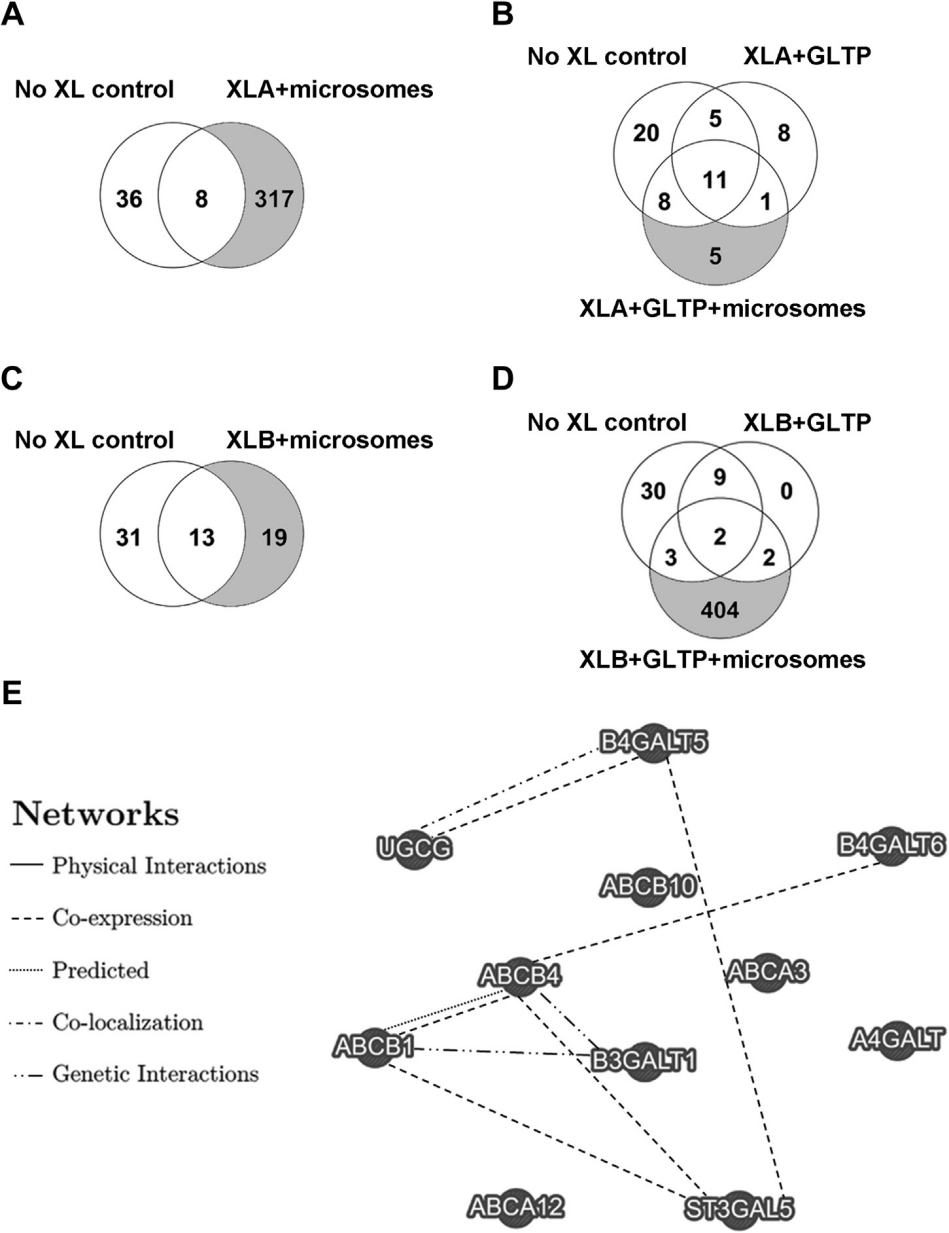
DU-145 microsomal proteins cross-linked by GlcCer photoprobes. XLA or XLB cross-linker preincubated ± GLTP were incubated with unfractionated microsomes at 37°C for 1 h in the dark before cross-linking with UV light for 15 min. After solubilization, samples were purified by streptavidin-coupled beads, denatured, digested, and analyzed by mass-spectrometry-based proteomics. The control sample (without cross-linker) was compared with (A) XLA cross-linked microsomal proteins, (B) XLA cross-linked GLTP, and GLTP delivered XLA cross-linked microsomal proteins, (C) XLB cross-linked microsomal proteins, and (D) XLB cross-linked GLTP, and GLTP delivered XLB cross-linked microsomal proteins. Three candidate flippases were identified, ABCA3, ABCB4, and ABCB10, along with β-1,3-galactosyltransferase 1 (B3GALT1), when XLB was delivered to unfractionated microsomes by GLTP. E: Networks between ABCB1, ABCA12, glycosyltransferases, and identified proteins were compared using GeneMANIA (www.genemania.org). Coexpression links two genes when expression levels are similar under different conditions. Predicted functional relationships between genes are indicated. Colocalization links genes that are both expressed in the same tissue or in the same cellular location. Genetic interactions link genes in which perturbation of one gene is modified by perturbation of the second gene. B3GALT1 has genetic interactions with ABCB1 and ABCB4. ABCB4 shows a predicted functional relationship with ABCB1 and is coexpressed with ST3GAL5 (β-galactoside α-2,3-sialyltransferase 5, GM_3_ synthase), B4GALT6 (β-1,4-galactosyltransferase 6, LacCer synthase), and ABCB1.

For identification of potential GlcCer flippases, GlcCer-based photoprobe stereoisomers XLA and XLB (see supplemental Fig. S1 in supplemental data for structure) were cross-linked in DU-145 cell unfractionated microsomes ± glycolipid transfer protein (GLTP) for cross-linker delivery and insertion into the microsomal membranes as previously (61). XLA and XLB were designed to mimic native GlcCer by maintaining the acyl group via native GlcCer fatty acid substitution with D, L-2-aminohexadecanoic acid, resulting in two diastereomers with different conformations at the stereogenic center circled in supplemental Fig. S1 (61). The 2-amino group was coupled to a cleavable photoreactive aryl azide, for protein cross-linking close to the GlcCer head group, and a biotin tag for protein isolation (61). Both XLA and XLB specifically cross-linked GLTP, a GlcCer-binding protein, but not other GSL-binding proteins (cholera and Shiga toxins) (61). GLTP cross-linking by XLA and XLB was inhibited by addition of GlcCer (61). Only XLB and not XLA was a LCS substrate; however, XLA inhibited fluorescent GlcCer analog nitrobenzoxadiazole (NBD)-GlcCer conversion to NBD-LacCer by LCS (61). DU-145 cells were chosen because of their exemplary acidic and neutral GSL content. Bound proteins were purified by streptavidin-coupled magnetic beads, denatured, digested, and identified by mass spectrometry. Control sample (without cross-linkers) was compared with cross-linked microsomal samples to determine relevant proteins (Fig. 1). There were 317 microsomal proteins cross-linked with XLA (Fig. 1A), but only five proteins cross-linked when GLTP delivered XLA (Fig. 1B). Nineteen proteins were identified when cross-linked with XLB (Fig. 1C), but 404 proteins were cross-linked with XLB when delivered with GLTP (Fig. 1D). Cross-linked GLTP was identified in all samples containing GLTP. In the XLB and GLTP sample, three ABC transporter candidate flippases were identified, ABCA3, ABCB4, and ABCB10, together with one bona fide GlcCer-binding protein, β-1,3-galactosyltransferase 1 (B3GALT1) (67). For information on protein and peptide identification, see supplemental Table S3 and supplemental Table S4. The list of proteins cross-linked when XLB was delivered with GLTP can also be found in supplemental data (supplemental Table S5). Relationships between identified proteins, ABCB1, ABCA12, and glycosyltransferases were determined using GeneMANIA (Fig. 1E). ABCB4 shows a predicted functional relationship with ABCB1 and is coexpressed with B4GALT6 (β-1,4-galactosyltransferase 6, LacCer synthase), ST3GAL5 (β-galactoside α-2,3-sialyltransferase 5, GM_3_ synthase), and ABCB1. B3GALT1 (Lc_4_ synthase), which was cross-linked, has genetic interactions with ABCB1 and ABCB4.

### siRNA screen in DU-145 cells

The putative GlcCer flippases identified by proteomics (ABCA3, ABCB4, ABCB10) were depleted by siRNA in DU-145 cells to investigate effects on GSL metabolism. Other protein knockdowns were also conducted, GCS, and suspected GlcCer translocases ABCB1 and ABCA12. Since not all siRNA sequences deplete gene expression equally, three siRNA sequences were made for each gene and screened for knockdown efficacy. siRNA was named by protein name and the position acted on in the gene sequence. DU-145 cells were transiently transfected with siRNA. mRNA was extracted, and analyzed by reverse transcription polymerase chain reaction (RT-PCR) amplification, and agarose gel electrophoresis. Negative control (NC) siRNA was provided as a scrambled sequence. GCS-833 and ABCB1-3323 knockdowns were most effective in DU-145 cells (supplemental Fig. S2A, F respectively) among other siRNA sequences (not shown). Of ABCA3-1006, ABCA3-1154, and ABCA3-4495, the most effective knockdown was ABCA3-1006 (supplemental Fig. S2B). ABCB4-980 knockdown was more effective than ABCB4-1447 and ABCB4-2046 (supplemental Fig. S2C). ABCB10-841 was the most effective between ABCB10-841, ABCB10-1039, and ABCB10-1506 (supplemental Fig. S2D). ABCA12-2663 depleted ABCA12 RNA more than ABCA12-667 and ABCA12-4771 (supplemental Fig. S2E).

### GCS depletion significantly reduced all GSLs

**Fig. 2:**
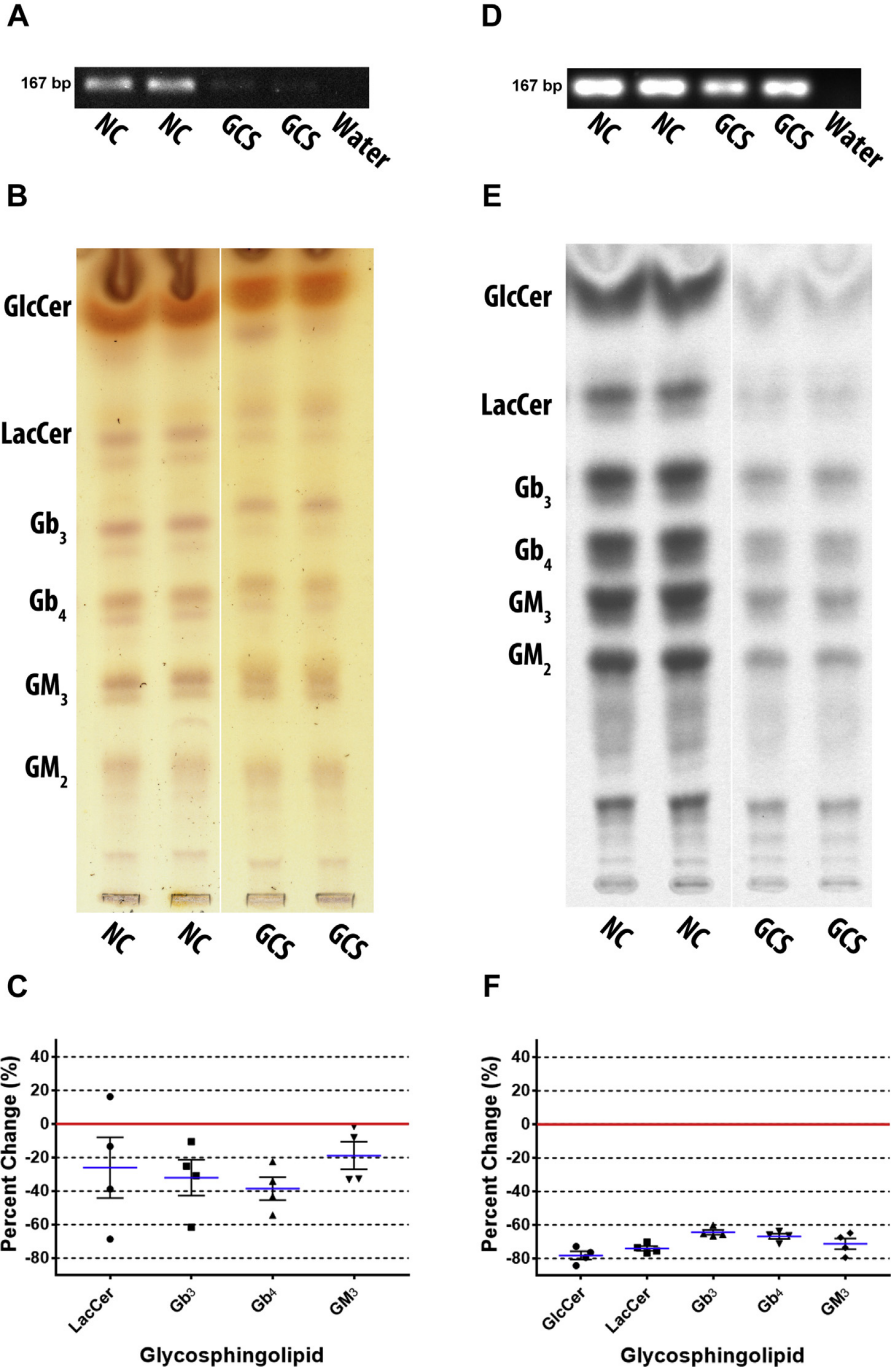
GCS knockdown reduced all GSLs. To estimate the maximum GSL depletion that could be achieved, GCS was depleted by siRNA in DU-145 cells. Total GSLs were examined post transfection, (A) representative image of GCS knockdown confirmed by RT-PCR with GSC primers (55°C annealing temperature, 25 cycles), (B) representative image of GSLs extracted and analyzed by TLC stained with orcinol (white line shows TLC plate splicing of samples run on the same plate), (C) scatter plot of quantified TLCs shows all GSLs are reduced (n = 2, in duplicate). Mean percent change represented as blue lines, standard error of mean represented as black solid lines, and each data point signified as circle for LacCer, square for Gb_3_, triangle for Gb_4_, and inverted triangle for GM_3_. DU-145 cells were metabolically labeled with ^14^C-galactose for 5 h post transfection, (D) representative image of GCS RNA transcript knockdown confirmed by RT-PCR (55°C annealing temperature, 30 cycles), (E) GSLs were extracted and newly synthesized GSLs were analyzed by TLC autoradiography (representative image; white line shows TLC plate splicing of samples run on the same plate), (F) scatter plot of quantified TLCs shows all GSLs are reduced, significantly more than total GSLs stained with orcinol (n = 2, in duplicate). Mean percent change represented as blue lines, standard error of mean represented as black solid lines, and each data point signified as circle for GlcCer, square for LacCer, triangle for Gb_3_, inverted triangle for Gb_4_, and diamond for GM_3_. The NC lanes in B are reused in Figs. 6B and 7B as they are from the same experiment/TLC separation. Similarly, the NC lanes in E are reused in Figs. 3E, 4E, and 5E.

GCS knockdown was a control to estimate the maximum possible GSL depletion. DU-145 cells were transfected with GCS-833 siRNA. Knockdown was confirmed by RT-PCR (Fig. 2A). GSLs were extracted and total GSL levels were analyzed by thin-layer chromatography (TLC) stained with orcinol (Fig. 2B). Quantified GSLs from TLC plates (Fig. 2C) show that LacCer was reduced by a mean of 26.1% ± 18.1, Gb_3_ by 31.9% ± 10.7, Gb_4_ by 38.5% ± 6.8, and GM_3_ by 18.8% ± 8.2. GlcCer could not be quantified due to interference from orcinol negative species. GM_2_ was not quantified due to inconsistent resolution.

GSLs stained with orcinol measured total GSL levels including residual pre-knockdown GSLs. To observe effects on GSL anabolism post knockdown exclusively, DU-145 cells were metabolically labeled with ^14^C-galactose for 5 h post transfection with GCS-833 siRNA. Figure 2D shows that GCS transcript was depleted by knockdown. GSLs were extracted, resolved by TLC, and newly synthesized GSLs detected by autoradiography (Fig. 2E). Quantified GSLs (Fig. 2F) show a mean reduction of GlcCer by 78.1% ± 2.4, LacCer by 73.9% ± 1.4, Gb_3_ by 64.4% ± 1.4, Gb_4_ by 66.8% ± 1.5, and GM_3_ by 71.2% ± 3.4. GM_2_ band appears reduced in representative TLC plate; however, it was not quantified due to inconsistent resolution.

Therefore, all GSLs were reduced in both GCS knockdown studies of total GSL and metabolically labeled GSL analyses. However, GSL differences between NC and GCS knockdown are significantly greater (approximately 2-fold) in metabolically labeled GSLs than total GSLs. Maximum (possible) inhibition for all GSLs was ∼70%.

### ABCA3 knockdown decreased complex GSLs, but increased LacCer

**Fig. 3:**
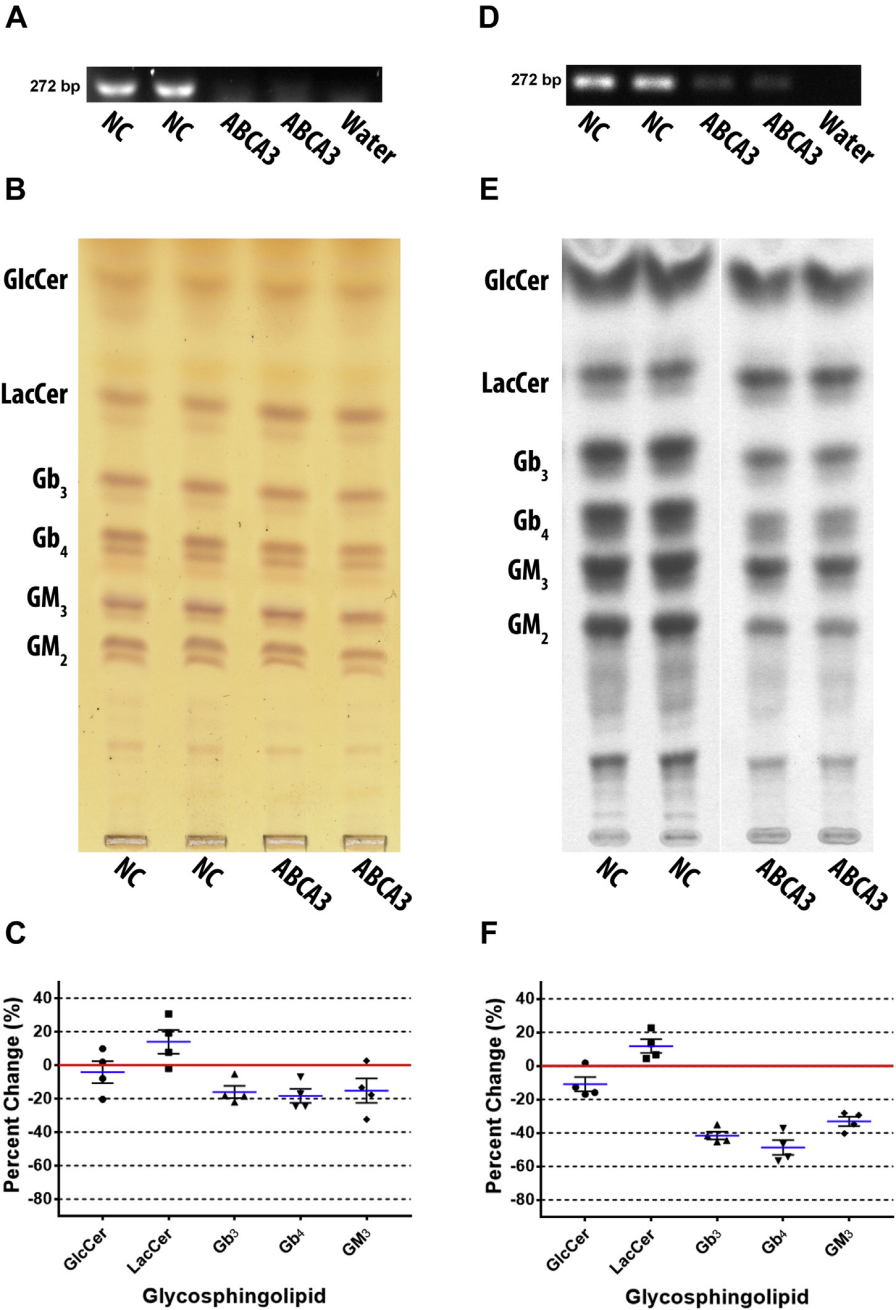
ABCA3 knockdown reduced complex GSLs but increased LacCer. ABCA3 was depleted by siRNA in DU-145 cells, and the effect on GSL biosynthesis examined post transfection, (A) ABCA3 RNA transcript reduction was confirmed by RT-PCR using ABCA3 primers with annealing temperature of 57°C and 30 cycles (representative image shown), (B) GSLs were extracted and analyzed by TLC stained with orcinol (representative TLC plate), (C) scatter plot of quantified TLCs shows LacCer is increased and complex GSLs are reduced (n = 2, in duplicate). Mean percent change represented as blue lines, standard error of mean represented as black solid lines, and each data point signified as circle for GlcCer, square for LacCer, triangle for Gb_3_, inverted triangle for Gb_4_, and diamond for GM_3_. Cells were metabolically labeled with ^14^C-galactose for 5 h post transfection, (D) knockdown was confirmed by RT-PCR using ABCA3 primers with annealing temperature of 57°C and 30 cycles (representative image), (E) GSLs were extracted and newly synthesized GSLs were analyzed by TLC autoradiography (representative TLC plate; white line shows TLC plate splicing of samples run on the same plate), (F) scatter plot of quantified TLCs show complex GSLs were reduced but LacCer was increased (n = 2, in duplicate). Mean percent change represented as blue lines, standard error of mean represented as black solid lines, and each data point signified as circle for GlcCer, square for LacCer, triangle for Gb_3_, inverted triangle for Gb_4_, and diamond for GM_3_. The NC lanes in B are reused in Figs. 4B and 5B as they are from the same experiment/TLC separation.

ABCA3 was depleted by ABCA3-1006 siRNA transfection in DU-145 cells to investigate its role in GSL biosynthesis. Knockdown was verified by RT-PCR (Fig. 3A). TLC separated GSLs were detected by orcinol stain (Fig. 3B). Quantified GSLs (Fig. 3C) show that GlcCer was reduced by mean of 4.1% ± 6.5, Gb_3_ by 16.0% ± 3.7, Gb_4_ by 18.26% ± 4.1, and GM_3_ by 15.2% ± 7.2, but LacCer was increased by 13.9% ± 7.0. GM_2_ was reduced but not quantified.

DU-145 cells were metabolically labeled with ^14^C-galactose for 5 h post transfection. RT-PCR shows that ABCA3 RNA was reduced by transient knockdown (Fig. 3D). GSLs were separated by TLC (Fig. 3E), and autoradiograms were quantified (Fig. 3F). GlcCer was reduced by a mean of 11.0% ± 4.3, Gb_3_ by 41.7% ± 2.3, Gb_4_ by 48.7% ± 4.4, and GM_3_ by 33.2% ± 2.8. In contrast, LacCer was increased by 11.8% ± 4.1. The representative TLC shows GM_2_ was reduced, but was not quantified.

In summary, ABCA3 depletion in DU-145 cells resulted in decreased Gb_3_, Gb_4_, GM_3_, but increased LacCer. All GSLs (except LacCer) were significantly more reduced when metabolically labeled.

### ABCB4 knockdown preferentially reduced neutral GSLs

**Fig. 4:**
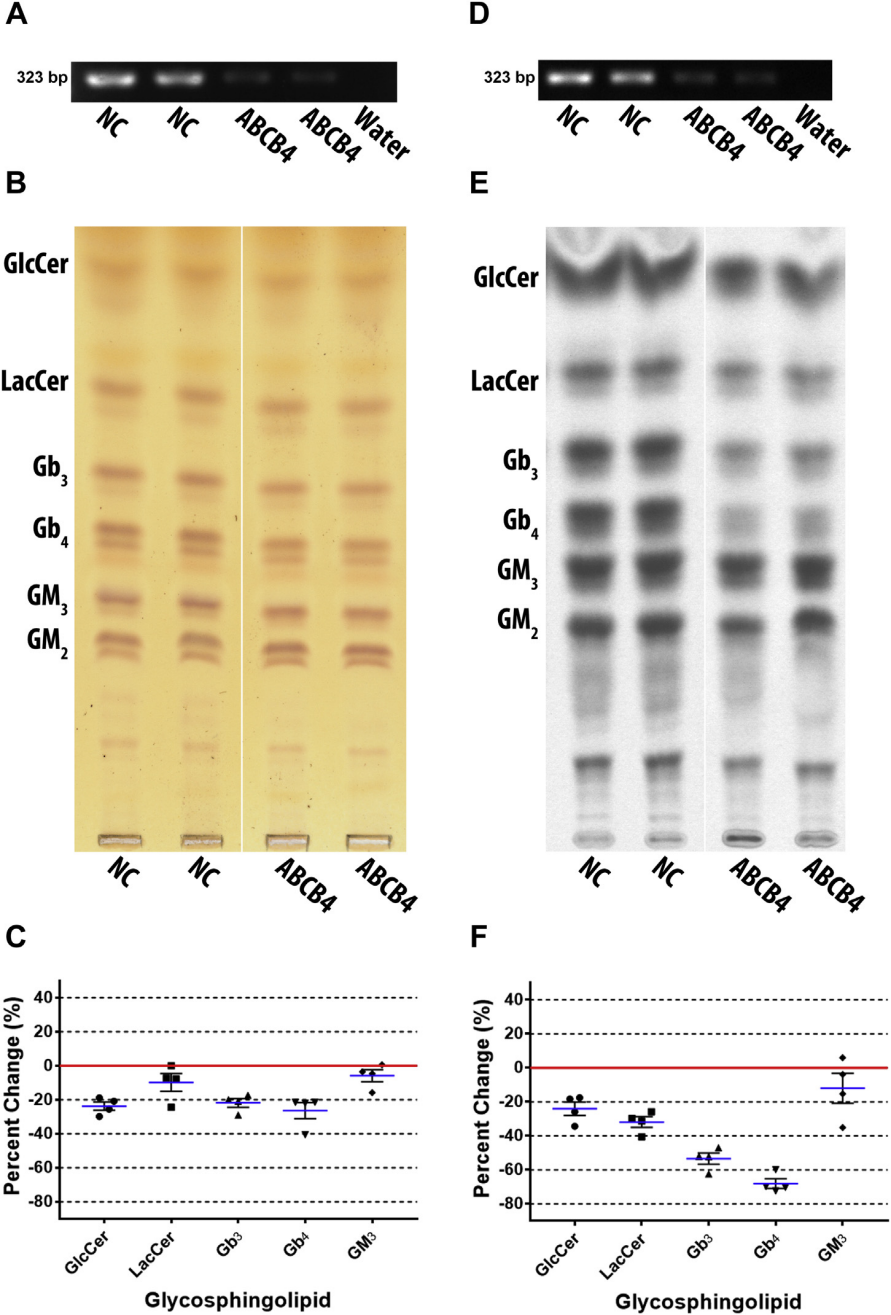
ABCB4 knockdown preferentially reduced neutral GSLs. ABCB4 was depleted by siRNA in DU-145 cells and the effect on GSL biosynthesis examined post transfection, (A) knockdown of ABCB4 RNA transcript was confirmed by RT-PCR using ABCB4 primers with an annealing temperature of 57°C and 35 cycles (representative image), (B) GSLs were extracted and analyzed by TLC stained with orcinol (representative TLC plate; white line shows TLC plate splicing of samples run on the same plate), (C) scatter plot of quantified GSLs shows neutral GSLs were preferentially reduced (n = 2, in duplicate). Mean percent change represented as blue lines, standard error of mean represented as black solid lines, and each data point signified as circle for GlcCer, square for LacCer, triangle for Gb_3_, inverted triangle for Gb_4_, and diamond for GM_3_. Cells were metabolically labeled with ^14^C-galactose for 5 h post transfection, (D) knockdown was confirmed by RT-PCR using ABCB4 primers with an annealing temperature of 57°C and 35 cycles (representative image), (E) GSLs were extracted and analyzed by TLC autoradiography (representative TLC plate; white line shows TLC plate splicing of samples run on the same plate), (F) scatter plot of quantified TLCs shows neutral GSLs were preferentially reduced (n = 2, in duplicate). Mean percent change represented as blue lines, standard error of mean represented as black solid lines, and each data point signified as circle for GlcCer, square for LacCer, triangle for Gb_3_, inverted triangle for Gb_4_, and diamond for GM_3_.

ABCB4 was depleted by ABCB4-980 siRNA transfection in DU-145 cells to determine its effects on GSL metabolism. Transfection was confirmed by RT-PCR (Fig. 4A). Extracted GSLs were separated by TLC and stained with orcinol (Fig. 4B). Quantified GSLs (Fig. 4C) show that ABCB4 depletion reduced GlcCer by a mean of 23.7% ± 2.5, LacCer by 9.8% ± 5.2, Gb_3_ by 21.8% ± 2.5, Gb_4_ by 26.4% ± 4.7, and GM_3_ by 5.8% ± 3.5. GM_2_ was not quantified; however, it does not appear reduced.

DU-145 cells were metabolically labeled with ^14^C-galactose for 5 h post transfection. Knockdown efficacy was confirmed by RT-PCR (Fig. 4D). GSLs were resolved by TLC (Fig. 4E), and autoradiograms were quantified (Fig. 4F). ABCB4 knockdown decreased GlcCer by a mean of 24.0% ± 3.9, LacCer by 31.8% ± 3.1, Gb_3_ by 53.4% ± 3.2, Gb_4_ by 68.1% ± 2.8, and GM_3_ by 12.1% ± 8.8. GM_2_ appears slightly reduced in the representative TLC plate.

ABCB4 depletion in DU-145 cells decreased all GSLs, but preferentially reduced neutral GSLs LacCer, Gb_3_, and Gb_4_. These changes were particularly evident in the metabolically labeled GSLs compared with total GSLs observed by orcinol stain.

### ABCB10 knockdown reduced both neutral and acidic GSLs

**Fig. 5:**
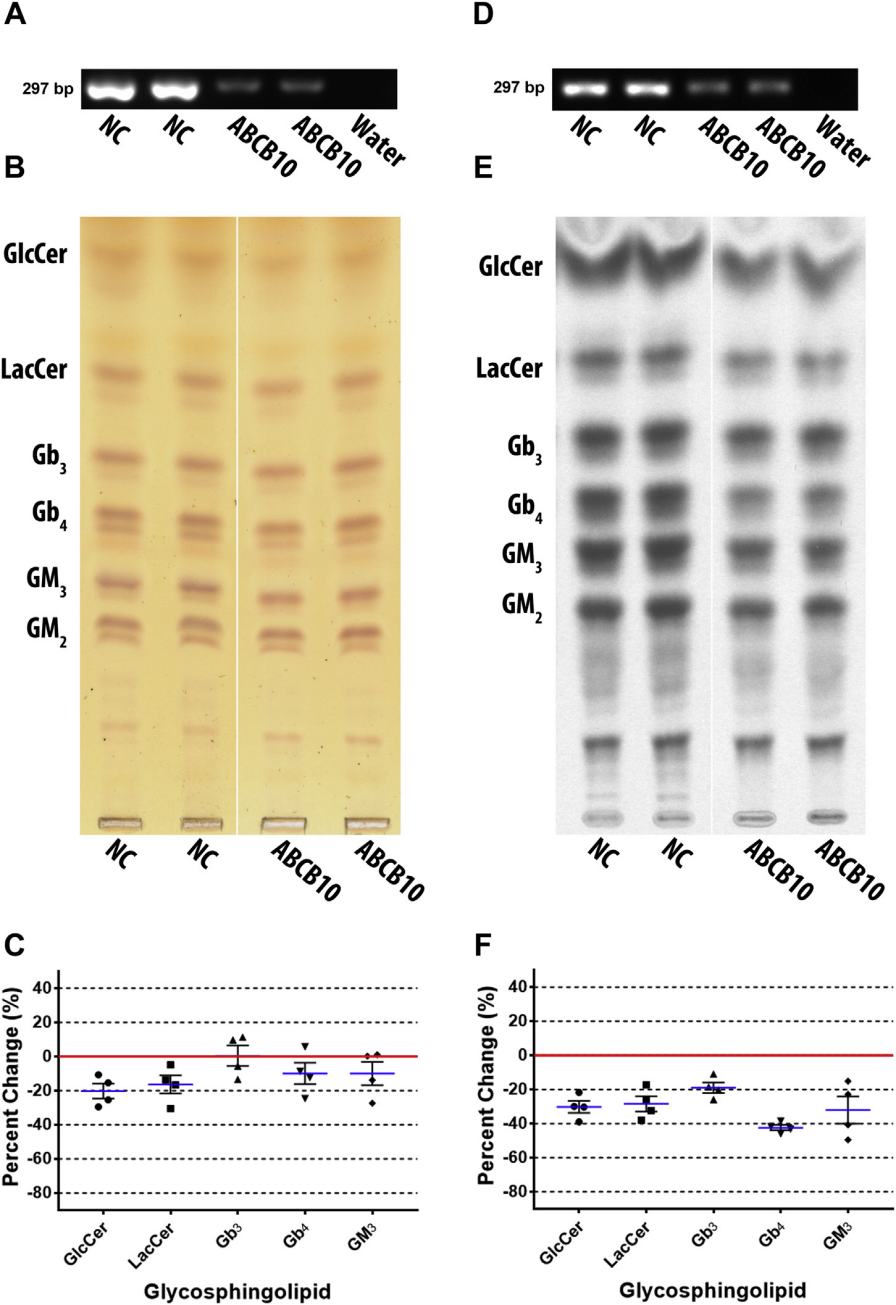
ABCB10 knockdown reduced all GSLs. ABCB10 was depleted by siRNA in DU-145 cells and the effect on GSL metabolism examined post transfection, (A) RT-PCR (ABCB10 primers with an annealing temperature of 57°C and 35 cycles) shows ABCB10 RNA transcript was reduced by knockdown (representative image), (B) GSLs were extracted and analyzed by TLC stained with orcinol (representative TLC plate; white line shows TLC plate splicing of samples run on the same plate), (C) scatter plot of quantified TLCs show GSLs are slightly reduced except for Gb_3_ (n = 2, in duplicate). Mean percent change represented as blue lines, standard error of mean represented as black solid lines, and each data point signified as circle for GlcCer, square for LacCer, triangle for Gb_3_, inverted triangle for Gb_4_, and diamond for GM_3_. Cells were metabolically labeled with ^14^C-galactose for 5 h post transfection, (D) knockdown was confirmed by RT-PCR with ABCB10 primers with an annealing temperature of 57°C and 30 cycles (representative image), (E) GSLs were extracted and newly synthesized GSLs were analyzed by TLC autoradiography (representative TLC plate; white line shows TLC plate splicing of samples run on the same plate), (F) scatter plot of quantified TLCs show all GSLs were reduced (least for Gb_3_) (n = 2, in duplicate). Mean percent change represented as blue lines, standard error of mean represented as black solid lines, and each data point signified as circle for GlcCer, square for LacCer, triangle for Gb_3_, inverted triangle for Gb_4_, and diamond for GM_3_.

ABCB10 was depleted by ABCB10-841 siRNA transfection in DU-145 cells to determine its role in GSL biosynthesis. Knockdown efficacy was observed by RT-PCR (Fig. 5A), which shows reduced ABCB10 mRNA transcript. GSLs were examined by TLC stained with orcinol (Fig. 5B). Quantified data show decreased GlcCer by a mean of 20.2% ± 4.4, LacCer by 16.4% ± 5.3, Gb_4_ by 9.9 ± 6.3, and GM_3_ by 10.0 ± 6.7, but Gb_3_ had a minor increase of 0.5% ± 6.0 (Fig. 5C). GM_2_ was not quantified; however, it appears slightly reduced in the representative TLC plate.

DU-145 cells were metabolically labeled with ^14^C-galactose for 5 h post transfection. ABCB10 mRNA transcript reduction was confirmed by RT-PCR (Fig. 5D). Radiolabeled GSLs were detected by TLC autoradiography (Fig. 5E). Quantified GSLs (Fig. 5F) revealed decreased GlcCer by a mean of 30.2% ± 3.5, LacCer by 28.4% ± 4.5, Gb_3_ by 19.0% ± 3.1, Gb_4_ by 42.3% ± 1.6, and GM_3_ by 32.0% ± 7.9. GM_2_ appears reduced in representative TLC plate, but was not quantified.

In summary, ABCB10 depletion in DU-145 cells decreased both neutral and acidic GSLs. However, these decreases were significantly greater in metabolically labeled GSLs than total GSLs.

### ABCA12 depletion preferentially reduced neutral GSL biosynthesis

**Fig. 6:**
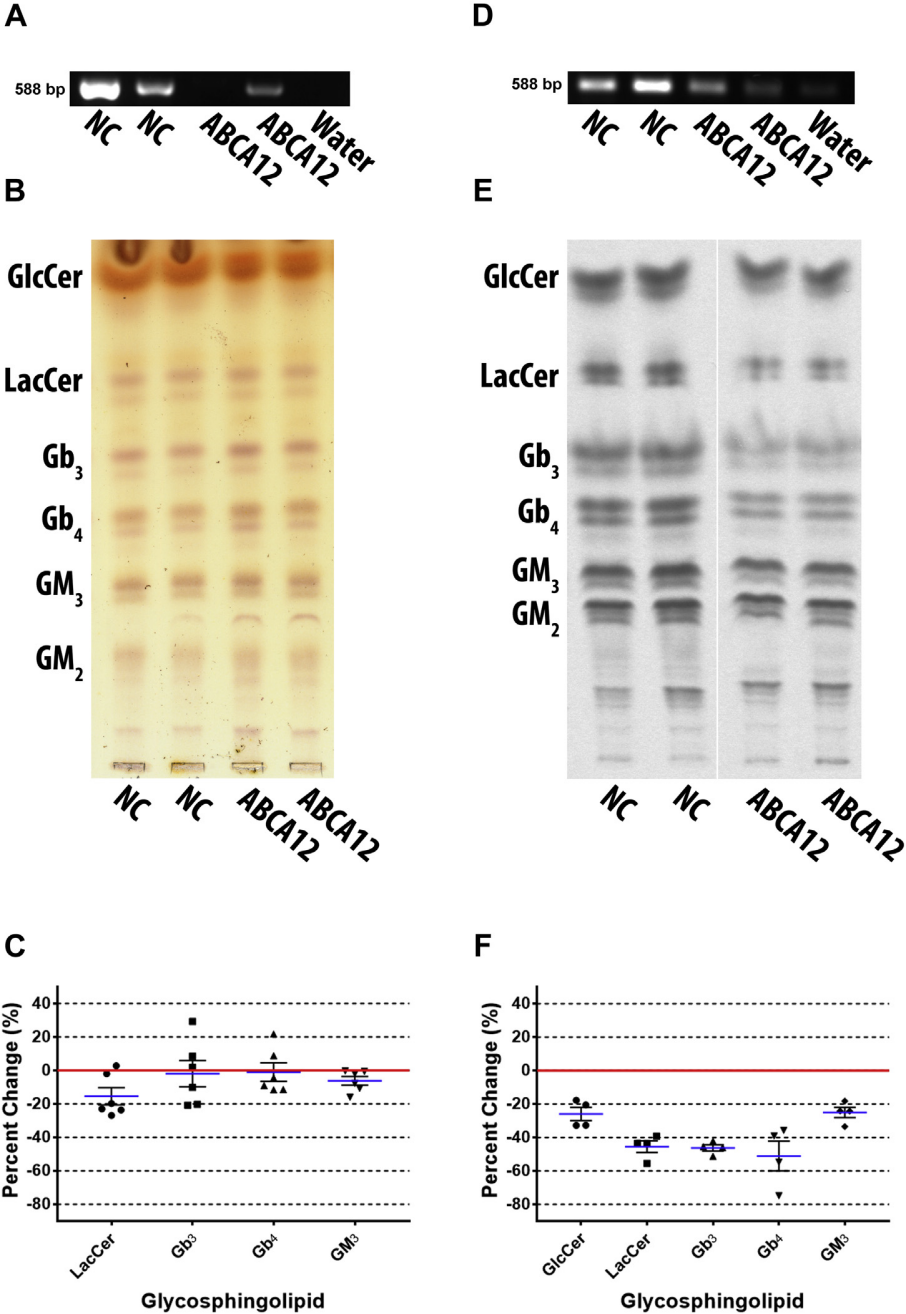
ABCA12 knockdown reduced all de novo GSL biosynthesis but neutral GSLs were preferentially affected. ABCA12 was depleted by siRNA in DU-145 cells to determine if ABCA12 is involved in GSL biosynthesis. Total GSLs were examined post transfection, (A) knockdown was confirmed by RT-PCR using ABCA12 primers with an annealing temperature of 55°C and 40 cycles (representative image), (B) GSLs were extracted and analyzed by TLC stained with orcinol (representative TLC plate), (C) scatter plot of quantified TLCs shows only LacCer is reduced (n = 3, in duplicate). Mean percent change represented as blue lines, standard error of mean represented as black solid lines, and each data point signified as circle for LacCer, square for Gb_3_, triangle for Gb_4_, and inverted triangle for GM_3_. Cells were metabolically labeled with ^14^C-galactose for 5 h post transfection, (D) RT-PCR (ABCA12 primers with an annealing temperature of 55°C and 37 cycles) was used to confirm ABCA12 RNA transcript depletion (representative image), (E) GSLs were extracted and newly synthesized GSLs were analyzed by TLC autoradiography (representative TLC plate; white line shows TLC plate splicing of samples run on the same plate), (F) scatter plot of quantified TLCs shows all GSLs were reduced with a preference for neutral GSLs (n = 2, in duplicate). Mean percent change represented as blue lines, standard error of mean represented as black solid lines, and each data point signified as circle for GlcCer, square for LacCer, triangle for Gb_3_, inverted triangle for Gb_4_, and diamond for GM_3_. The NC lanes in E are reused in Fig. 7E as they are from the same experiment/TLC separation.

ABCA12 has been shown to transport GlcCer across membranes of lamellar bodies in keratinocytes (57); however, its role in GSL biosynthesis has not been examined. Therefore, we transiently knocked down ABCA12 in DU-145 cells to determine its role in GSL metabolism. Cells were transfected with ABCA12-2663 siRNA, and ABCA12 mRNA depletion was confirmed by RT-PCR (Fig. 6A). GSLs were observed by orcinol-stained TLC (Fig. 6B) and quantified (Fig. 6C). ABCA12 knockdown reduced LacCer by a mean of 15.4% ± 5.1, GM_3_ by 6.2% ± 2.6, and negligible decreased Gb_3_ by 1.9% ± 7.9, and Gb_4_ by 1.0% ± 5.5. GlcCer was not quantified due to orcinol negative band interference. GM_2_ was also not quantified due to inconsistent resolution; however, it appears unchanged in the representative TLC.

DU-145 cells were metabolically labeled with ^14^C-galactose for 5 h post transfection. ABCA12 mRNA depletion was confirmed by RT-PCR (Fig. 6D). Newly synthesized GSLs were analyzed by TLC autoradiography (Fig. 6E). Quantified GSLs (Fig. 6F) show that GlcCer was reduced by a mean of 25.9% ± 4.0, LacCer by 45.4% ± 3.5, Gb_3_ by 46.2% ± 1.9, Gb_4_ by 54.1% ± 9.0, and GM_3_ by 25.0% ± 3.2. GM_2_ appears to be reduced in representative TLC plate, but was not quantified.

In summary, ABCA12 knockdown changes in GSL levels were minimal when total GSLs were analyzed. However, neutral GSLs were preferentially reduced when the cells were metabolically labeled.

### ABCB1 knockdown preferentially reduced gangliosides but increased Gb_3_

**Fig. 7:**
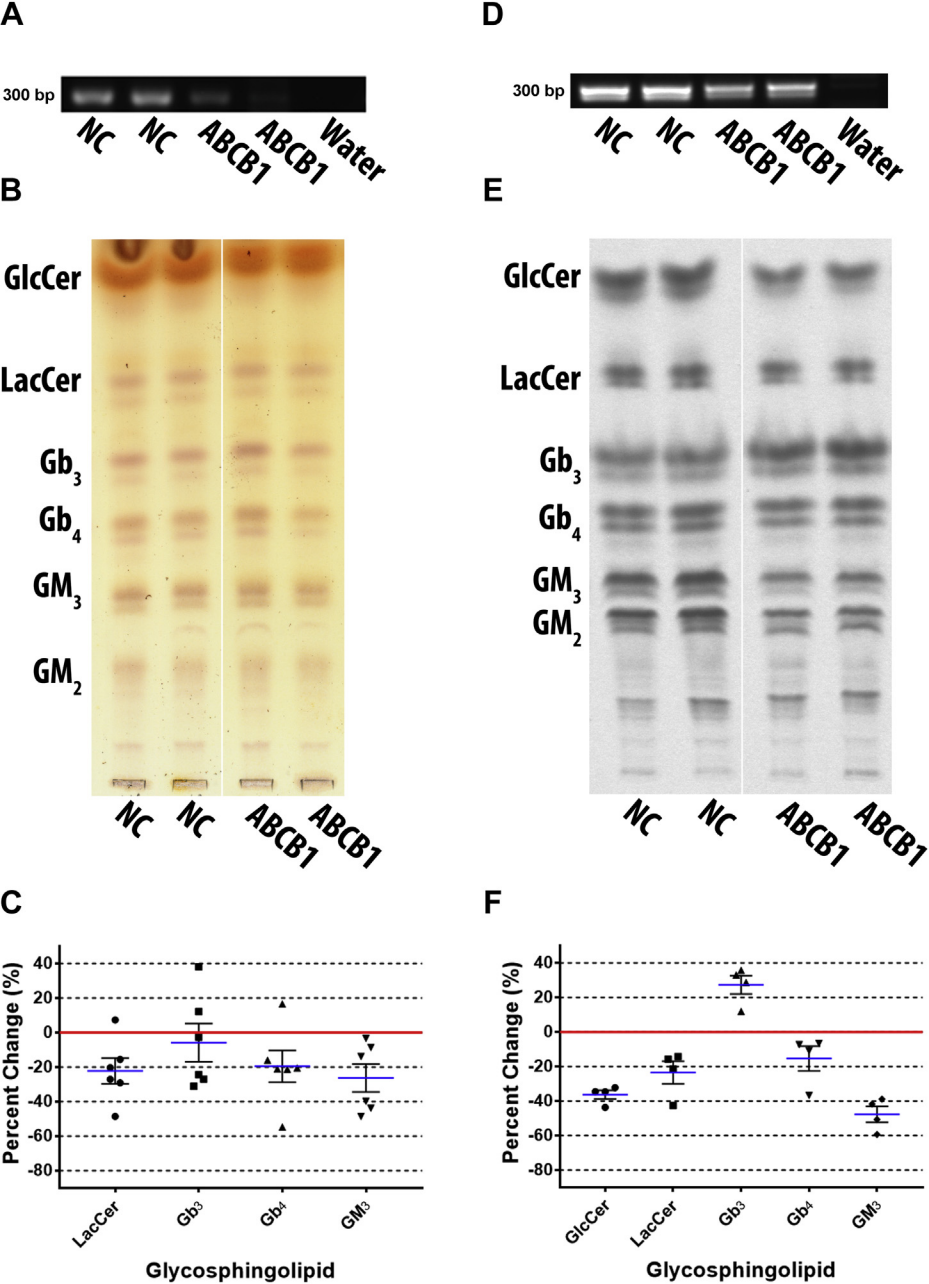
ABCB1 knockdown preferentially reduced gangliosides but increased Gb_3_. ABCB1 was depleted by siRNA in DU-145 cells, to clarify ABCB1 involvement in GSL biosynthesis. Total GSLs were examined post transfection, (A) knockdown was confirmed by RT-PCR using ABCB1 primers with an annealing temperature of 60°C and 40 cycles (representative image), (B) GSLs were extracted and analyzed by TLC stained with orcinol (representative TLC plate; white line shows TLC plate splicing of samples run on the same plate), (C) scatter plot of quantified TLCs shows all GSLs are reduced (n = 3, in duplicate). Mean percent change represented as blue lines, standard error of mean represented as black solid lines, and each data point signified as circle for LacCer, square for Gb_3_, triangle for Gb_4_, and inverted triangle for GM_3_. Cells were metabolically labeled with ^14^C-galactose for 5 h post transfection, (D) RT-PCR (ABCB1 primers with an annealing temperature of 60°C and 40 cycles) shows knockdown effectively reduced ABCB1 mRNA transcript (representative image), (E) extracted GSLs were analyzed by TLC autoradiography (representative TLC plate; white line shows TLC plate splicing of samples run on the same plate), (F) scatter plot of quantified TLCs shows GM_3_ was preferentially reduced but Gb_3_ was increased (n = 2, in duplicate). Mean percent change represented as blue lines, standard error of mean represented as black solid lines, and each data point signified as circle for GlcCer, square for LacCer, triangle for Gb_3_, inverted triangle for Gb_4_, and diamond for GM_3_.

ABCB1 is currently the only transporter proposed to flip GlcCer across the Golgi membrane for complex GSL biosynthesis. To elucidate the specific role of ABCB1 in GSL biosynthesis, it was knocked down in DU-145 cells by ABCB1-3323 siRNA transfection. Depletion of ABCB1 transcript was verified by RT-PCR (Fig. 7A). Total GSLs resolved by TLC were detected by orcinol (Fig. 7B) and quantified (Fig. 7C). LacCer was decreased by a mean of 22.2% ± 7.5, Gb_3_ by 5.8% ± 11.1, Gb_4_ by 19.5% ± 9.2, GM_3_ by 26.3% ± 8.1. GlcCer was not quantified due to an interfering orcinol negative species. GM_2_ was also not quantified due to inconsistent resolution; however, it appears somewhat reduced in the representative TLC plate.

To examine the effect of ABCB1 knockdown on newly synthesized GSLs DU-145 cells were metabolically labeled with ^14^C-galactose for 5 h post transfection. RT-PCR shows that ABCB1 mRNA was reduced by knockdown with ABCB1-3323 siRNA (Fig. 7D). GSLs were detected by TLC autoradiography (Fig. 7E) and quantified (Fig. 7F). GlcCer was decreased by a mean of 36.2% ± 2.5, LacCer by 23.5% ± 6.5, Gb_4_ by 15.3% ± 7.2, and GM_3_ by 47.6% ± 4.6, but Gb_3_ increased by 27.4% ± 5.3. GM_2_ appears reduced in the representative TLC plate, but was not quantified.

Both total GSLs and metabolically labeled GSLs have similar results for LacCer, GM_3_, and Gb_4_. Metabolic data shows significant increase in Gb_3_, while total GSL data shows insignificant decrease. GM_3_ is substantially more decreased in metabolic data. Overall, ABCB1 knockdown preferentially decreased GM_3_ but increased Gb_3_.

Changes in Gb3 levels post ABC transporter knockdowns were not acyl chain selective.

To examine if ABC transporters preferentially transport GlcCer with specific acyl chain compositions, Gb_3_ content was analyzed by mass spectrometry post ABC knockdowns. Depletion of ABCA3, ABCB1, ABCB4, or ABCB10 resulted in no preferential change in Gb_3_ species (supplemental Fig. S3).

## Discussion

We previously designed GlcCer analog photoprobes XLA and XLB (diastereomers) to mimic native GlcCer and isolate GlcCer binding proteins (61). The GlcCer acyl group was substituted with D, L-2-aminohexadecanoic acid, allowing for the coupling of a trifunctional cross-linker sulfo-N-hydroxysuccinimidyl-2-(6-[biotinamido]-2-(p-azido benzamido)-hexanoamido) ethyl-1,3′-dithioproprionate (sulfo-SBED) to the 2-amino group while maintaining the GlcCer fatty acid moiety (61). This produced diastereomers with different acyl chain conformations and facilitated the addition of a cleavable photoreactive aryl azide for target protein cross-linking and a biotin tag for protein isolation (61). XLA and XLB specificity for GlcCer-binding proteins among other GSL-binding proteins was validated; however, only XLB acted as a substrate for LCS, while XLA acted as an inhibitor (61). In this study, the probes were only used as a screen to identify potential GlcCer-binding proteins, while involvement in GSL biosynthesis would be confirmed through knockdown studies.

We previously demonstrated GLTP enhancement of (luminal) microsomal GSL synthesis, which was amplified by ATP, consistent with GLTP delivery of cytosolic nascent GlcCer to an ATP-dependent step (ATP-dependent flippase) (61). GLTP was also established as a cytosolic GSL transporter in model membranes (68, 69). Therefore, we used GLTP to deliver and insert our GlcCer analog cross-linkers into DU-145 cell unfractionated microsomal membranes to identify GlcCer binding proteins and putative undiscovered GlcCer flippases. DU-145 cell line was selected for this study due to their extensive GSL profile containing both neutral and acidic GSLs. This provides a means to investigate whether different GSL pathways are mediated by distinct flippases. Three ABC transporter candidate flippases, ABCA3, ABCB4, and ABCB10, were identified by mass spectrometry only when DU-145 microsomal proteins were cross-linked by GLTP delivered XLB. B3GALT1 (Lc_4_ synthase), which is a downstream glycosyltransferase in GSL synthesis (67), was also identified. Since XLB could be converted into its LacCer form in unfractionated microsomes (61), it is feasible to cross-link downstream glycosyltransferases with XLB (61). Furthermore, B3GALT1 has been shown to use GlcCer as a substrate in addition to Lc_3_ (67).

Major differences between the XLA and XLB proteomics results were observed. This is the direct result of these cross-linkers being diastereomers containing the fatty acid moieties in different orientations (61). XLA and XLB precursors (2A-GlcCer A and B respectively derived from coupling a D,L 2-aminohexadecanoic acid mixture) ran very differently on TLC due to a 2A-GlcCer A intramolecular hydrogen bond between the primary amine of the fatty acid moiety and hydroxyl group of the sphingosine moiety (61). Thus conformation of fatty acid stereoisomers alone can have a major impact on GSL polarity. Even after 2A-GlcCer A conversion to XLA (primary amine conversion to amide linkage), XLA was more hydrophilic than XLB. This was supported by the significant number of XLA cross-linked microsomal proteins when delivered as micelles compared with XLB. XLA was better able to incorporate into microsomal membranes as micelles than via GLTP delivery. GSL acyl structure is key for GLTP binding (70). The XLA acyl chain orientation appears to prevent or slow its off-rate from GLTP, resulting in cross-linked GLTP and a significantly lower number of cross-linked microsomal proteins compared with XLB under the same conditions. This is supported by previous data showing XLA was not converted to its LacCer analog in unfractionated microsomes (unlike XLB), due to restricted access of the glucose 4′OH required for LCS action, yet XLA still inhibited NBD-LacCer synthesis from NBD-GlcCer (61). XLA fatty acid chain orientation could prevent its translocation or interaction with GlcCer flippases since flippase activity could be acyl chain conformation selective. Furthermore, XLA had a lower affinity for GLTP than XLB (61). The lack of LCS, ABCB1, or ABCA12 cross-linking may be due to a lower XLB affinity for these proteins compared with native GlcCer, in addition to their low protein expression (ABCA12 and ABCB1 required high cycling for RT-PCR suggesting low mRNA levels, Figs. 6, 7) in DU-145 cells. One method to ameliorate this would be to deplete or inhibit GCS to reduce endogenous GlcCer before cross-linking. However, this could potentially reduce expression of GlcCer flippases, since GCS expression has been shown to correlate with ABCB1 expression (48).

Candidate flippases were selected because they are in the same protein family as ABCB1, which has known GlcCer flippase activity (71), and many family members are lipid flippases (72). However, none of these cross-linked candidates have been previously shown to interact with GlcCer or be involved with other GSLs.

ABCA3 plays a role in pulmonary surfactant formation by transporting phospholipids into lamellar bodies (73, 74). Lung surfactant is a mixture of cholesterol, phospholipids, and surfactant protein formed and stored in lamellar bodies of lung alveolar type II pneumocytes until secretion (73). Mutations in ABCA3 result in rare lung disorders (74, 75). Interestingly, lung surfactant protein SP-A binds glucosylceramide and other neutral GSLs (76); therefore GlcCer may play a role in surfactant formation through translocation into lamellar bodies by ABCA3.

ABCB4 has been suggested as a phospholipid floppase, which flops PC from the inner to the outer leaflet of the canalicular membrane, making PC available for extraction into bile (43). Defects in ABCB4 cause intrahepatic cholestasis (77). ABCB4 (also known as MDR3) is closely related to ABCB1 (78% identity (78, 79, 80)), and like ABCB1, can act as a drug efflux pump (81) contributing to tumor drug resistance (80, 82). Interestingly, ABCB4 is also implicated in glucose metabolism (83), and GSLs play a key role in diabetes (84).

ABCB10 is a δ-aminolevulinic acid transporter (85), in the inner mitochondrial membrane involved in heme synthesis and oxidative stress protection (86). The location of the nucleotide-binding domains and conformation suggests that it transports substrates out of the mitochondrial matrix (87, 88). Interestingly, ganglioside GD3 acts as an apoptotic regulator by interacting with mitochondria and recruiting apoptotic pathways (89). GM_1_-accumulation at GSL-enriched microdomains in mitochondrial-associated ER membranes may influence Ca^2+^-mediated apoptotic signaling (90). Diabetic heart tissue has increased mitochondrial LacCer, with decreased calcium retention capacity and respiration, suggesting LacCer as the primary sphingolipid responsible for mitochondrial defects (91). Therefore, ABCB10 may play a role in GD3, GM_1_, and/or LacCer regulation.

GCS, ABCB1, ABCA12, and ABC transporters identified by mass spectrometry were depleted in DU-145 cells. Cell growth and cell viability were constant for negative control (scrambled sequence), ABC transporters, and GCS knockdowns. GSLs post-KD were loaded equally to TLC plates based on cell number and analyzed in two different ways, metabolic labeling or orcinol stain. Metabolic labeling assesses de novo GSL biosynthesis, while orcinol detection of TLC separated GSLs gives an index of total GSL content, the sum of anabolism, catabolism, and trafficking. DU-145 cells metabolically labeled with ^14^C-galactose produced radiolabeled GlcCer due to conversion of UDP-galactose to UDP-glucose by UDP-galactose 4-epimerase (92). DU-145 cells have little to no detectable GalCer (93), further confirmed by the GCS KD showing ∼80% reduction of GlcCer; therefore GlcCer and GalCer were not separated via borate impregnated TLC.

GCS knockdown was used as a positive control to determine the maximal extent of GSL depletion. All GSLs were more significantly reduced when GSLs were metabolically labeled than when total content was stained with orcinol. This was also true for all ABC transporter KDs, especially ABCA12. The reduction of GSL synthesis may itself alter GSL trafficking (94, 95) and hence lysosomal degradation.

All three putative GlcCer flippases identified by proteomics markedly affected GSL biosynthesis when depleted by siRNA transfection. ABCA3 knockdown reduced higher-order GSL synthesis but increased LacCer production, possibly the result of increased GlcCer availability to a LacCer synthase where subsequent anabolic glycosyltransferases are not present. ABCB4 knockdown preferentially reduced globo-series GSLs as opposed to gangliosides. This suggests it is involved in the nonvesicular GSL glycosylation pathway, where it may translocate FAPP2 delivered GlcCer across the TGN membrane, for globo-series synthesis (36). ABCB10 depletion reduced neutral and acidic GSLs. This suggests that ABCB10 is involved in both vesicular and nonvesicular GSL glycosylation pathways and may translocate GlcCer at both the *cis*-Golgi and the TGN.

ABCA12 and ABCB1 were not identified in the proteomics results; however, they have been shown to interact with GlcCer (42, 58). ABCA12 knockdown reduced LacCer but did not show a significant change in Gb_3_, Gb_4_, or GM_3_ when total GSLs were analyzed. However, when the cells were metabolically labeled, neutral GSLs were preferentially reduced. These differences between total and metabolic results could be due to compensatory feedback mechanisms resisting changes in GSL levels by slowing or restricting the rate of GSL catabolism when de novo GSL biosynthesis is inhibited. ABCB1 knockdown preferentially reduced gangliosides and decreased neutral GSLs except for Gb_3_, which increased. Increased Gb_3_ suggests increased GlcCer availability for a Gb_3-_specific pathway unavailable to Gb_4_ synthase. This is similar to the effect of fumonisin on GSL synthesis (96); the major metabolite of fumonisin is aminopentol, which is an ABCB1 substrate (97). These ABCB1 knockdown results are contrary to published data where ABCB1 inhibition with cyclosporin A decreased LacCer and Gb_3_ synthesis (47). However, this may be due to inhibition of ABCB4 by cyclosporin A (98) rather than ABCB1. Overexpression of ABCB1 in Madin-Darby canine kidney cells resulted in increased GlcCer, LacCer, and Gb_3_ (45), and ovarian cancer cells that overexpress ABCB1 have increased GlcCer (no change in GCS expression) and lower levels of LacCer and gangliosides (99). Overexpression could force ABCB1 localization to the TGN in addition to the Golgi cisternae, as shown with GM_3_ synthase overexpression (36). It is noteworthy that these findings were from a different cell line than the DU-145 cells used in our knockdown studies, suggesting the role of ABC transporters in GSL biosynthesis within the Golgi/TGN may be tissue and/or cell type dependent. Mouse mdr1a/b knockdown results in decreased GSL levels in skin fibroblasts, but brain GSLs are unaltered (100), further indicating a cell-type-dependent role of ABCB1 in GSL synthesis. In cross-breeding of Fabry and ABCB1 knockout mice, we found that only the heart and spleen showed reduced GlcCer, whereas LacCer was generally elevated in these tissues and Gb_3_ was not affected (101). It has been argued that lack of a clear phenotype for MDR knockout mice is not consistent with a physiological function (102); however, we show in this study that there is a marked overlap and redundancy for ABC proteins in GSL biosynthesis, possibly as GlcCer flippases.

DU-145 cells are a hypotriploid cell line characterized by several genetic deletions (103, 104, 105). None of these deletions include the ABC proteins in this study, though ABCA12 contains a silent mutation, and ABCB1 and ABCA3 contain missense mutations (106). It is unknown whether these missense mutations could affect GlcCer transport or protein localization.

**Fig. 8:**
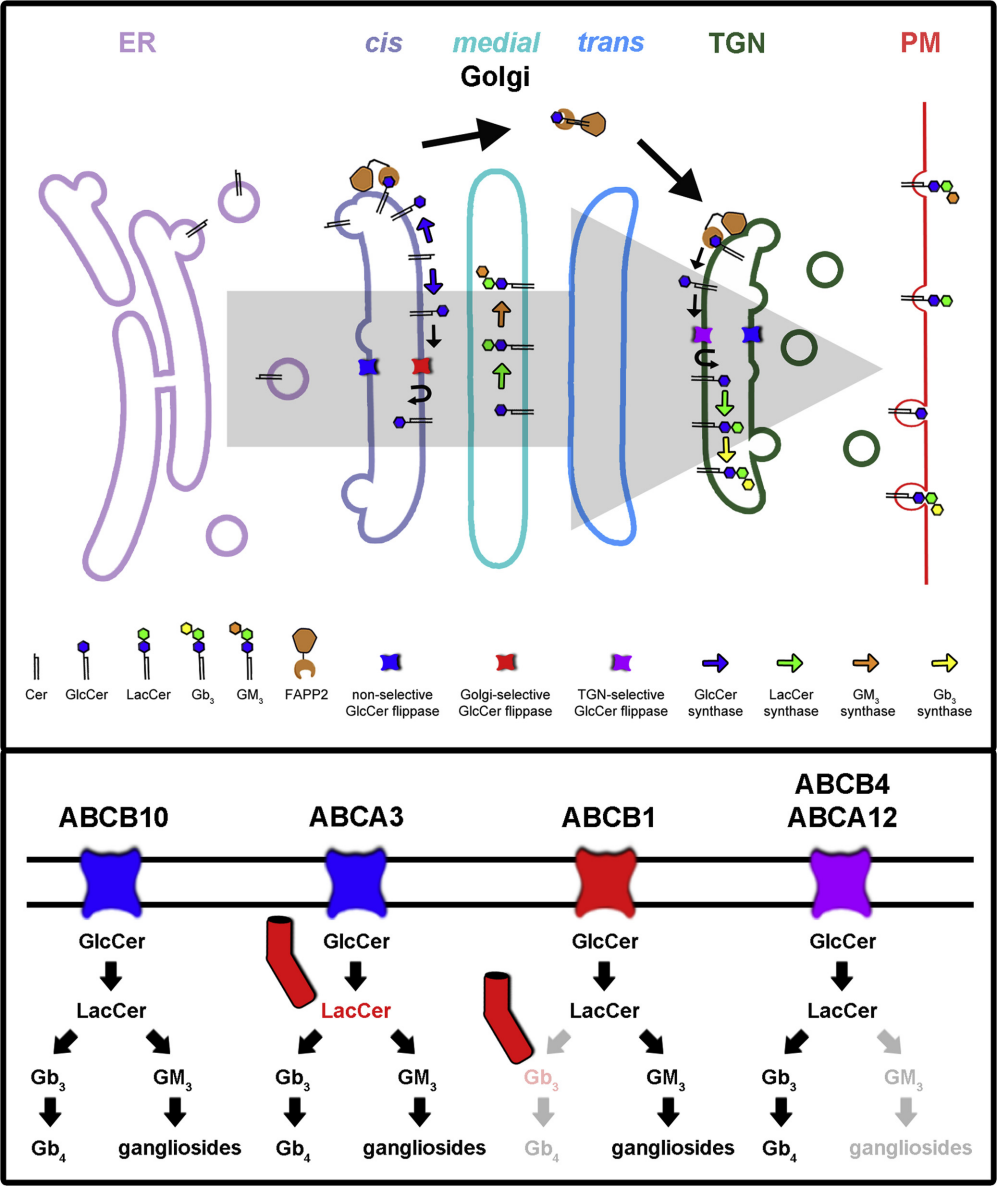
Schematic of GSL synthesis and metabolic channeling in DU-145 cells. GlcCer synthesized on the cytosolic leaflet of the Golgi (mainly *cis*/*medial*-Golgi) can follow two pathways, a FAPP2-independent vesicular pathway or a FAPP2-dependent non-vesicular pathway (upper panel). For ganglioside GSL synthesis, GlcCer can be translocated by Golgi-selective flippases to the lumen and transported through the Golgi cisternae and TGN via vesicular transport. For neutral GSL synthesis GlcCer is transported to the TGN by FAPP2, where it is translocated to the lumen by TGN-selective flippases. The gray arrow in the upper panel shows the direction of vesicular transport. The divergent effects of ABC depletion in DU-145 cells on select GSLs show complex GSLs are synthesized in a step-wise fashion from separate pools of GlcCer (lower panel). ABCB10 knockdown results in the loss of both neutral GSLs and gangliosides. Therefore, GlcCer flipped by this transporter must be available for both ganglioside and globo-series GSL synthesis. Depletion of ABCA3 reduced all GSLs except LacCer was increased. Thus, ABCA3 counteracts the metabolic channeling (represented by red text and cylindrical channel) of a GlcCer, and subsequent LacCer, pool which is unavailable to Gb_3_ or GM_3_ synthase. Knockdown of ABCB1 reduced all GSLs (preferentially GM_3_) except Gb_3_ was increased. Therefore, ABCB1 is a Golgi-selective GlcCer flippase involved in GM_3_ synthesis but not Gb_3_ synthesis (represented by decreased opacity), and counteracts a GlcCer/LacCer channeled source for the synthesis of Gb_3_ (represented by red text and cylindrical channel) unavailable to Gb_4_ synthase. ABCB4 and ABCA12 knockdowns preferentially reduced globo-series GSL levels. Thus, ABCB4 and ABCA12 are TGN-selective GlcCer flippases, which provides metabolic channeling for globo-series GSL synthesis, by generating a GlcCer pool that is not available for GM_3_ synthase (represented by decreased opacity).

GSL synthesis is restricted by glycosyltransferases sub-Golgi localization, but does not follow a straightforward pathway from the *cis* to *trans*-Golgi to synthesize complex GSLs (36, 107). Knockdown of ABC transporters in DU-145 cells differentially affected GSL biosynthesis, possibly as GlcCer Golgi flippases, suggesting separate and overlapping Golgi pools of GlcCer for differential GSL synthesis. We propose an expansion of the current model (2). GlcCer synthesized on the cytosolic leaflet of the *cis*-Golgi can follow two pathways (Fig. 8). For ganglioside GSL synthesis, GlcCer can be translocated by Golgi-selective flippases to the lumen and transported through the Golgi cisternae and TGN via vesicular transport to the cell surface. For globo-series GSL synthesis GlcCer is transported to the TGN by FAPP2, where it is translocated to the lumen by TGN-selective flippases. ABCB4 and ABCA12 may be TGN-selective flippases (globo-series pathway), while ABCA3 and ABCB10 may be nonselective flippases. ABCB1 may be a Golgi-selective flippase (gangliosides pathway) in DU-145 cells specifically. However, final proof of native GlcCer flippase function must await ABC membrane reconstitution studies. Nevertheless, it is clear that ABC transporters appear to be differentially involved in GSL biosynthesis.

We (108) and others (109, 110) have shown that the fatty acid component of membrane GSLs modulates their receptor function; therefore, it may also affect ABC GlcCer flippase activity. Mass spectrometry of a representative GSL (Gb_3_) after each ABC transporter knockdown showed no change in fatty acid content, consistent with no ABC flippase GlcCer fatty acid preference.

The fact that ABC depletion can increase certain GSLs means their presence can suppress certain otherwise operative GSL biosynthetic pathways. These results are consistent with metabolic substrate channeling (111), whereby the product of one enzyme reaction, in this case, at a potential branch point, is directly transferred to only one of several possible next enzymes in a given pathway. Several such glycosyltransferase complexes in GSL biosynthesis have been described (112, 113, 114). Figure 8 shows a schematic of potential ABC anabolic channels in DU-145 cell Golgi cisternae/TGN. Some GlcCer, and subsequently LacCer, pools are not available for ganglioside biosynthesis, whereas other pools are not used for globo-series GSL synthesis. In addition, some LacCer is not available for downstream GSL synthesis (counteracted by ABCA3), and Gb_3_ can be made without access to Gb_4_ synthase, from a separate channelled LacCer source (counteracted by ABCB1). The distinction between ganglioside and globo-series GSL precursor LacCer pools may extend to other GSL series and relate to distinct changes in GSL series during differentiation (115).

It is possible that non-GSL ABC transporter substrates (e.g., brefeldin A (116), fumonisin (97), PS (117) for ABCB1, PC for ABCB4 (43), ABCA3 (118)) could affect GSL biosynthesis. Cooperative multiple drug binding by ABCB1 to promote drug transport (119, 120) may provide a kinetic basis. Substrates can induce or stimulate ABCB1 efflux (121). Endogenous/exogenous substrates for ABCB1 (122) (or other ABC transporters) could therefore provide an unsuspected means to adjust cellular GSL biosynthesis. In this regard, if Gb_3_, like its adamantyl derivative (123), is an ABCB1 inhibitor, this would provide a feedback regulation of GSL synthesis, which would serve to amplify relative Gb_3_ levels.

ABC transporter expression is increased in cancer cells (124) and varies widely between cell/tissue types (106, 125). ABC transporter expression levels in DU-145 cells vary (ABCA12 < ABCB4 < ABCB1 < ABCA3 < ABCB10) (106), which may be a contributing factor for specific and redundant ABC transporter involvement in acid/neutral GSL pathways. Therefore, the role of ABC transporters in GSL biosynthesis could vary according to cell/tissue.

Nascent ABC transporters destined for different subcellular compartments (e.g., ABCB10 to mitochondria, ABCA3 to lysosomes) are made in the ER and could function as GlcCer flippases during their glycosylation and anterograde transit through the Golgi. This suggests that additional regulatory mechanisms may exist during this transit. We have found that statins (which inhibit HMG Co-A reductase to reduce cholesterol synthesis and protein prenylation) markedly increase cellular GlcCer and Lc_3_ synthesis and alter Golgi GCS distribution (93). Many ABC transporters have been found to bind cholesterol (126), and several are involved in its transport (127, 128). Cholesterol-binding motifs are found in several ABC transporters, including ABCB1 (129). Cholesterol and GSLs accumulate in membrane lipid rafts (130) which contain ABC transporters (131). The cholesterol/GSL complex results in a GSL carbohydrate conformational change from membrane perpendicular to parallel (132), which masks the carbohydrate from ligand binding (133). Since there is a membrane cholesterol gradient from the ER to the TGN (134), this increase in concentration may differentially affect the relative sugar conformation of GlcCer, in addition to the energy barrier for membrane translocation within different Golgi regions, which may effect differential ABC transporter usage. ABC transporter cholesterol redistribution could in addition play a role in GSL biosynthetic control.

Metabolic channeling in GSL biosynthesis provides an opportunity for the regulation of an individual GSL or GSL subsets, rather than the total ablation of GSL synthesis, which results from the current use of inhibitors of GlcCer synthase (135, 136). ABC transporters and possibly ATPases may be potential GlcCer flippases and should be further investigated with membrane reconstitution of each recombinant transporter. Nevertheless, our discovery that multiple ABC transporters differentially control GSL biosynthesis provides potential targets for drug therapy to regulate specific GSL species or subsets in select cells/organs and thereby more precisely ameliorate pathology in GSL dysregulated diseases.

## Data availability

The raw mass spectrometric data has been deposited in MassIVE. To view dataset's webpage, go to https://doi.org/10.25345/C5RN31, Username: MSV000086365_reviewer, Password: LingwoodLab. To view dataset's files, go to ftp://massive.ucsd.edu/MSV000086365/, Username: MSV000086365, Password: LingwoodLab. All remaining data are contained within the article.

## Supplemental data

This article contains supplemental data (61, 66).

## Conflict of interest

The authors declare that they have no conflicts of interest with the contents of this article.
