# Supplementary material for: ATP-binding cassette transporters mediate differential biosynthesis of glycosphingolipid species
Source: J Lipid Res. 2021 Sep 28;62:100128. doi: 10.1016/j.jlr.2021.100128 (PMC8569594; doi:10.1016/j.jlr.2021.100128)
Supplement: Supplemental Fig. S1–S3 — and Tables S1, S2, S4, and S5 [file mmc1.docx]

**Supplemental data**

**ATP-binding cassette transporters mediate differential biosynthesis of glycosphingolipid species**

Monique Budani^1,2^, Christiane Auray-Blais^3^ and Clifford Lingwood^1,2,4,*^

^1^Division of Molecular Medicine, Research Institute, Hospital for Sick Children, Toronto, Ontario, Canada; ^2^Department of Laboratory Medicine & Pathobiology, University of Toronto, Toronto, Ontario, Canada; ^3^Division of Medical Genetics, Department of Pediatrics, Faculty of Medicine and Health Sciences, Université de Sherbrooke, Sherbrooke, Québec, Canada; ^4^Department of Biochemistry, University of Toronto, Toronto, Ontario, Canada; ^*^For correspondence: cling@sickkids.on.ca

**Table S1: siRNA sequences.**

| Gene: | Sense sequence (5’-3’): | Anti-sense sequence (5’-3’): |
| --- | --- | --- |
| Negative Control (NC) | UUC UCC GAA CGU GUC ACG UTT | ACG UGA CAC GUU CGG AGA ATT |
| ABCA3-1006 | GGG CAC UUG UGA UCA ACA UTT | AUG UUG AUC ACA AGU GCC CTT |
| ABCA3-1154 | GGC GGU GAA AUA UCA CCU ATT | UAG GUG AUA UUU CAC CGC CTT |
| ABCA3-4495 | GCA GUU UCU ACG AGA ACU ATT | UAG UUC UCG UAG AAA CUG CTT |
| ABCB4-980 | GGU AUU GCC UUC CUG UUA ATT | UUA ACA GGA AGG CAA UAC CTT |
| ABCB4-1447 | GCA GGA UAU UAG GAA CUU UTT | AAA GUU CCU AAU AUC CUG CTT |
| ABCB4-2046 | GCU GGA AAU CUC GCC UAU UTT | AAU AGG CGA GAU UUC CAG CTT |
| ABCB10-841 | GCA CAG GAG AAU UGA UUA ATT | UUA AUC AAU UCU CCU GUG CTT |
| ABCB10-1039 | GGC GAU AUC UAC GGA AAC UTT | AGU UUC CGU AGA UAU CGC CTT |
| ABCB10-1506 | CAG GGU GCU UUG GAG UUU ATT | UAA ACU CCA AAG CAC CCU GTT |
| ABCB1-3323 | CAC CCA GGC AAU GAU GUA UTT | AUA CAU CAU UGC CUG GGU GTT |
| ABCA12-667 | CCG GAA CAU AUA CUU UCA ATT | UUG AAA GUA UAU GUU CCG GTT |
| ABCA12-2663 | CCC AGU CAC AAA GGC AAU ATT | UAU UGC CUU UGU GAC UGG GTT |
| ABCA12-4771 | GGG AUG UUA UAU CCA AGA ATT | UUC UUG GAU AUA ACA UCC CTT |
| GCS-833 | CCA CCU UAG AGC AGG UAU ATT | UAU ACC UGC UCU AAG GUG GTT |

**Table S2: Primer sequences.**

| Gene: | Forward (5’-3’): | Reverse (5’-3’): | Product Size (bp): |
| --- | --- | --- | --- |
| ABCA3 | GTC GTG CAG GAG AAG GAA AG | TTG GCT TTG CTG AAG AAG GT | 272 |
| ABCB4 | CCA CAG CGA ACT GAT GAA GA | TGG CAA TGG CAC ATA CTG TT | 323 |
| ABCB10 | CCA AGC TGC CTT TTA ACG AG | TCC CAA TTT TGG ATG TCA GC | 297 |
| ABCA12 | AGA CCT ACA CAC GGT ACG GA | TAG GCT TCG GGG AGA TGT GA | 588 |
| ABCB1 | CAT TGG TGT GGT GAG TCA GG | ACC ACT GCT TCG CTT TCT GT | 300 |
| GCS | ATT CCA GAT ACG CTT ACT GAC AT | AAA CCA GTT ACA TTG GCA GAG AT | 167 |

**Table S3: Protein identification of ABCB4, ABCA3, ABCB10, and B3GALT1.**

| Protein name | Isoform 2 of Phosphatidylcholine translocator ABCB4 OS=Homo sapiens GN=ABCB4 | ATP-binding cassette sub-family A member 3 OS=Homo sapiens GN=ABCA3 PE=1 SV=2 | ATP-binding cassette sub-family B member 10, mitochondrial OS=Homo sapiens GN=ABCB10 PE=1 SV=2 | Beta-1,3-galactosyltransferase 1 OS=Homo sapiens GN=B3GALT1 PE=2 SV=1 |
| --- | --- | --- | --- | --- |
| Biological sample name | **XLB+GLTP+microsomes** | **XLB+GLTP+microsomes** | **XLB+GLTP+microsomes** | **XLB+GLTP+microsomes** |
| Protein accession numbers | **P21439-2\|MDR3_HUMAN,P21439-3\|MDR3_HUMAN,P21439\|MDR3_HUMAN** | **Q99758\|ABCA3_HUMAN** | **Q9NRK6\|ABCBA_HUMAN** | **Q9Y5Z6\|B3GT1_HUMAN** |
| Database sources | **Uniprot-Human-Nov12015.fasta** | **Uniprot-Human-Nov12015.fasta** | **Uniprot-Human-Nov12015.fasta** | **Uniprot-Human-Nov12015.fasta** |
| Protein molecular weight (Da) | **135,264.20** | **191,368.70** | **79,150.90** | **37,994.40** |
| Protein identification probability | **99.70%** | **99.70%** | **99.70%** | **99.70%** |
| Exclusive unique peptide count | **1** | **1** | **1** | **1** |
| Exclusive unique spectrum count | **1** | **1** | **1** | **1** |
| Exclusive spectrum count | **7** | **1** | **1** | **7** |
| Percentage of total spectra | **0.11%** | **0.02%** | **0.02%** | **0.11%** |
| Percentage sequence coverage | **0.86%** | **1.12%** | **1.49%** | **3.37%** |
| Peptide sequence | **QRIAIARALIR** | **YNIQYQENFYAWSAPGVGR** | **QRIAIARALLK** | **LLKPSTKPRRR** |
| Previous amino acid | **K** | **K** | **K** | **K** |
| Next amino acid | **Q** | **F** | **N** | **Y** |
| Best Peptide identification probability | **99.70%** | **99.70%** | **99.70%** | **99.70%** |
| Best Sequest deltaCn | **-0.111** | **-0.428** | **-0.546** | **-0.731** |
| Best Sequest XCorr | **0.031** | **0.316** | **0.228** | **0.025** |
| Best Sequest Sp | **0** | **0** | **0** | **0** |
| Best Sequest SpRank | **1** | **1** | **1** | **1** |
| Best Sequest Peak Ratio | **0** | **0** | **0** | **0** |
| Number of identified +1H spectra | **0** | **0** | **0** | **0** |
| Number of identified +2H spectra | **7** | **1** | **1** | **7** |
| Number of identified +3H spectra | **0** | **0** | **0** | **0** |
| Number of identified +4H spectra | **0** | **0** | **0** | **0** |
| Number of enzymatic termini | **2** | **2** | **2** | **2** |
| Calculated +1H Peptide Mass (AMU) | **1,280.83** | **2,266.01** | **1,253.81** | **1,351.86** |
| Median Retention Time | **2,675.45** | **3,072.57** | **3,587.30** | **705.867** |
| Total TIC | **8,725.13** | **392.69** | **9,355.57** | **10,010.30** |
| Peptide start index | **1134** | **1287** | **640** | **191** |
| Peptide stop index | **1144** | **1305** | **650** | **201** |

**Table S4: Peptide identification of B3GALT1, ABCB4, ABCA3, and ABCB10.**

| Protein name | Protein accession numbers | Sequence | Prob | SEQUEST XCorr | SEQUEST deltaCn | X! Tandem | NTT | Modifications | Observed | Actual Mass | Charge | Delta Da | Delta PPM | Stop | Retention Time | # Other Proteins | Intensity | Other Proteins | TIC | Spectrum ID | Start |
| --- | --- | --- | --- | --- | --- | --- | --- | --- | --- | --- | --- | --- | --- | --- | --- | --- | --- | --- | --- | --- | --- |
| Beta-1,3-galactosyltransferase 1 OS=Homo sapiens GN=B3GALT1 PE=2 SV=1 | **Q9Y5Z6\|B3GT1_HUMAN** | **(K)LLKPSTKPRRR(Y)** | **100%** | **0.0250358** | **-0.7313721** |  | **2** |  | **676.4336** | **1,350.85** | **2** | **-0.004735** | **-3.503** | **201** | **739.873** | **0** |  |  | **2,373.22** | **Monique-7-1968-2298_2298** | **191** |
| Beta-1,3-galactosyltransferase 1 OS=Homo sapiens GN=B3GALT1 PE=2 SV=1 | **Q9Y5Z6\|B3GT1_HUMAN** | **(K)LLKPSTKPRRR(Y)** | **100%** | **0.05547765** | **-0.6618376** |  | **2** |  | **676.4329** | **1,350.85** | **2** | **-0.006078** | **-4.496** | **201** | **705.867** | **0** |  |  | **1,581.02** | **Monique-7-1875-2190_2190** | **191** |
| Beta-1,3-galactosyltransferase 1 OS=Homo sapiens GN=B3GALT1 PE=2 SV=1 | **Q9Y5Z6\|B3GT1_HUMAN** | **(K)LLKPSTKPRRR(Y)** | **100%** | **0.0619486** | **-0.65320706** |  | **2** |  | **676.4337** | **1,350.85** | **2** | **-0.004613** | **-3.412** | **201** | **42.7501** | **0** |  |  | **2,121.37** | **Monique-7-116-136_136** | **191** |
| Beta-1,3-galactosyltransferase 1 OS=Homo sapiens GN=B3GALT1 PE=2 SV=1 | **Q9Y5Z6\|B3GT1_HUMAN** | **(K)LLKPSTKPRRR(Y)** | **100%** | **0.0952719** | **-0.27785543** |  | **2** |  | **676.4331** | **1,350.85** | **2** | **-0.005712** | **-4.225** | **201** | **1,024.78** | **0** |  |  | **855.24** | **Monique-7-2711-3166_3166** | **191** |
| Beta-1,3-galactosyltransferase 1 OS=Homo sapiens GN=B3GALT1 PE=2 SV=1 | **Q9Y5Z6\|B3GT1_HUMAN** | **(K)LLKPSTKPRRR(Y)** | **100%** | **0.1022433** | **-0.49210015** |  | **2** |  | **676.4342** | **1,350.85** | **2** | **-0.003514** | **-2.6** | **201** | **205.125** | **0** |  |  | **1,000.30** | **Monique-7-525-615_615** | **191** |
| Beta-1,3-galactosyltransferase 1 OS=Homo sapiens GN=B3GALT1 PE=2 SV=1 | **Q9Y5Z6\|B3GT1_HUMAN** | **(K)LLKPSTKPRRR(Y)** | **100%** | **0.1256078** | **-0.116514556** |  | **2** |  | **676.4316** | **1,350.85** | **2** | **-0.008763** | **-6.483** | **201** | **1,172.57** | **0** |  |  | **277.892** | **Monique-7-3034-3556_3556** | **191** |
| Beta-1,3-galactosyltransferase 1 OS=Homo sapiens GN=B3GALT1 PE=2 SV=1 | **Q9Y5Z6\|B3GT1_HUMAN** | **(K)LLKPSTKPRRR(Y)** | **100%** | **0.14899005** | **-0.20742644** |  | **2** |  | **676.4338** | **1,350.85** | **2** | **-0.004247** | **-3.141** | **201** | **396.092** | **0** |  |  | **1,801.27** | **Monique-7-1035-1210_1210** | **191** |
| Isoform 2 of Phosphatidylcholine translocator ABCB4 OS=Homo sapiens GN=ABCB4 | **P21439-2\|MDR3_HUMAN,P21439-3\|MDR3_HUMAN,P21439\|MDR3_HUMAN** | **(K)QRIAIARALIR(Q)** | **100%** | **0.03095365** | **-0.11076015** |  | **2** |  | **640.9219** | **1,279.83** | **2** | **0.009149** | **7.143** | **1191** | **3,295.40** | **0** |  |  | **259.019** | **Monique-7-5832-7789_7789** | **1181** |
| Isoform 2 of Phosphatidylcholine translocator ABCB4 OS=Homo sapiens GN=ABCB4 | **P21439-2\|MDR3_HUMAN,P21439-3\|MDR3_HUMAN,P21439\|MDR3_HUMAN** | **(K)QRIAIARALIR(Q)** | **100%** | **0.05838935** | **-0.23055439** |  | **2** |  | **640.9194** | **1,279.82** | **2** | **0.004145** | **3.236** | **1191** | **840.341** | **0** |  |  | **1,063.68** | **Monique-7-2237-2612_2612** | **1181** |
| Isoform 2 of Phosphatidylcholine translocator ABCB4 OS=Homo sapiens GN=ABCB4 | **P21439-2\|MDR3_HUMAN,P21439-3\|MDR3_HUMAN,P21439\|MDR3_HUMAN** | **(K)QRIAIARALIR(Q)** | **100%** | **0.07699155** | **-0.5893124** |  | **2** |  | **640.92** | **1,279.83** | **2** | **0.005365** | **4.189** | **1191** | **3,243.95** | **0** |  |  | **393.619** | **Monique-7-5758-7681_7681** | **1181** |
| Isoform 2 of Phosphatidylcholine translocator ABCB4 OS=Homo sapiens GN=ABCB4 | **P21439-2\|MDR3_HUMAN,P21439-3\|MDR3_HUMAN,P21439\|MDR3_HUMAN** | **(K)QRIAIARALIR(Q)** | **100%** | **0.11783885** | **-0.12015029** |  | **2** |  | **640.9221** | **1,279.83** | **2** | **0.009394** | **7.334** | **1191** | **1,667.07** | **0** |  |  | **526.907** | **Monique-7-3780-4619_4619** | **1181** |
| Isoform 2 of Phosphatidylcholine translocator ABCB4 OS=Homo sapiens GN=ABCB4 | **P21439-2\|MDR3_HUMAN,P21439-3\|MDR3_HUMAN,P21439\|MDR3_HUMAN** | **(K)QRIAIARALIR(Q)** | **100%** | **0.12451065** | **-0.43940035** |  | **2** |  | **640.921** | **1,279.83** | **2** | **0.007196** | **5.618** | **1191** | **3,515.80** | **0** |  |  | **3,186.78** | **Monique-7-6334-8408_8408** | **1181** |
| Isoform 2 of Phosphatidylcholine translocator ABCB4 OS=Homo sapiens GN=ABCB4 | **P21439-2\|MDR3_HUMAN,P21439-3\|MDR3_HUMAN,P21439\|MDR3_HUMAN** | **(K)QRIAIARALIR(Q)** | **100%** | **0.1523226** | **-0.34373143** |  | **2** |  | **640.9207** | **1,279.83** | **2** | **0.006586** | **5.142** | **1191** | **2,675.45** | **0** |  |  | **844.674** | **Monique-7-4886-6477_6477** | **1181** |
| Isoform 2 of Phosphatidylcholine translocator ABCB4 OS=Homo sapiens GN=ABCB4 | **P21439-2\|MDR3_HUMAN,P21439-3\|MDR3_HUMAN,P21439\|MDR3_HUMAN** | **(K)QRIAIARALIR(Q)** | **100%** | **0.2097222** | **-0.20207351** |  | **2** |  | **640.9203** | **1,279.83** | **2** | **0.005854** | **4.57** | **1191** | **41.6436** | **0** |  |  | **2,450.46** | **Monique-7-114-133_133** | **1181** |
| ATP-binding cassette sub-family A member 3 OS=Homo sapiens GN=ABCA3 PE=1 SV=2 | **Q99758\|ABCA3_HUMAN** | **(K)YNIqYqEnFYAWSAPGVGR(F)** | **100%** | **0.31561026** | **-0.42807704** |  | **2** | **Deamidated (+1), Deamidated (+1), Deamidated (+1)** | **1,133.52** | **2,265.02** | **2** | **0.01601** | **7.066** | **1305** | **3,072.57** | **0** |  |  | **392.69** | **Monique-7-5467-7290_7290** | **1287** |
| ATP-binding cassette sub-family B member 10, mitochondrial OS=Homo sapiens GN=ABCB10 PE=1 SV=2 | **Q9NRK6\|ABCBA_HUMAN** | **(K)qRIAIARALLK(N)** | **100%** | **0.2283424** | **-0.5458306** |  | **2** | **Deamidated (+1)** | **627.412** | **1,252.81** | **2** | **0.01146** | **9.139** | **650** | **3,587.30** | **0** |  |  | **9,355.57** | **Monique-7-6513-8628_8628** | **640** |

**Table S5: Proteins cross-linked with XLB when delivered with GLTP.**

See attached Excel file.

**
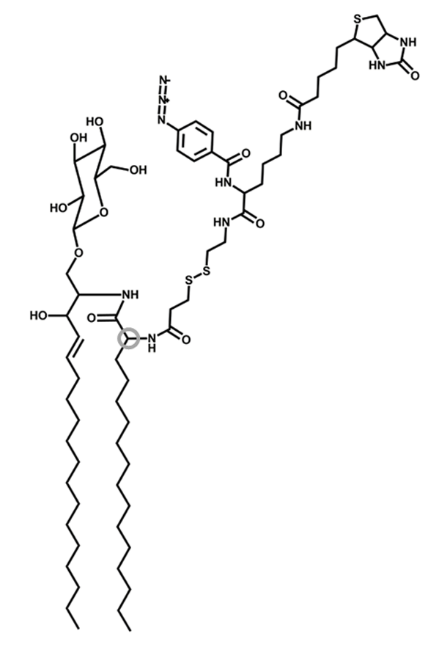
**

**Figure S1: Structure of GlcCer-based cross-linkers XLA and XLB**

XLA and XLB sterioisomer cross-linkers were constructed as previously described ([1](#_ENREF_1)) by coupling deacyl GlcCer to 2-aminohexodecanoic acid (D and L mixture) and the disulfide containing biotinylated photoactivable crosslinker, Sulfo-SBED. XLA and XLB differ at one stereogenic center circled in gray.

**
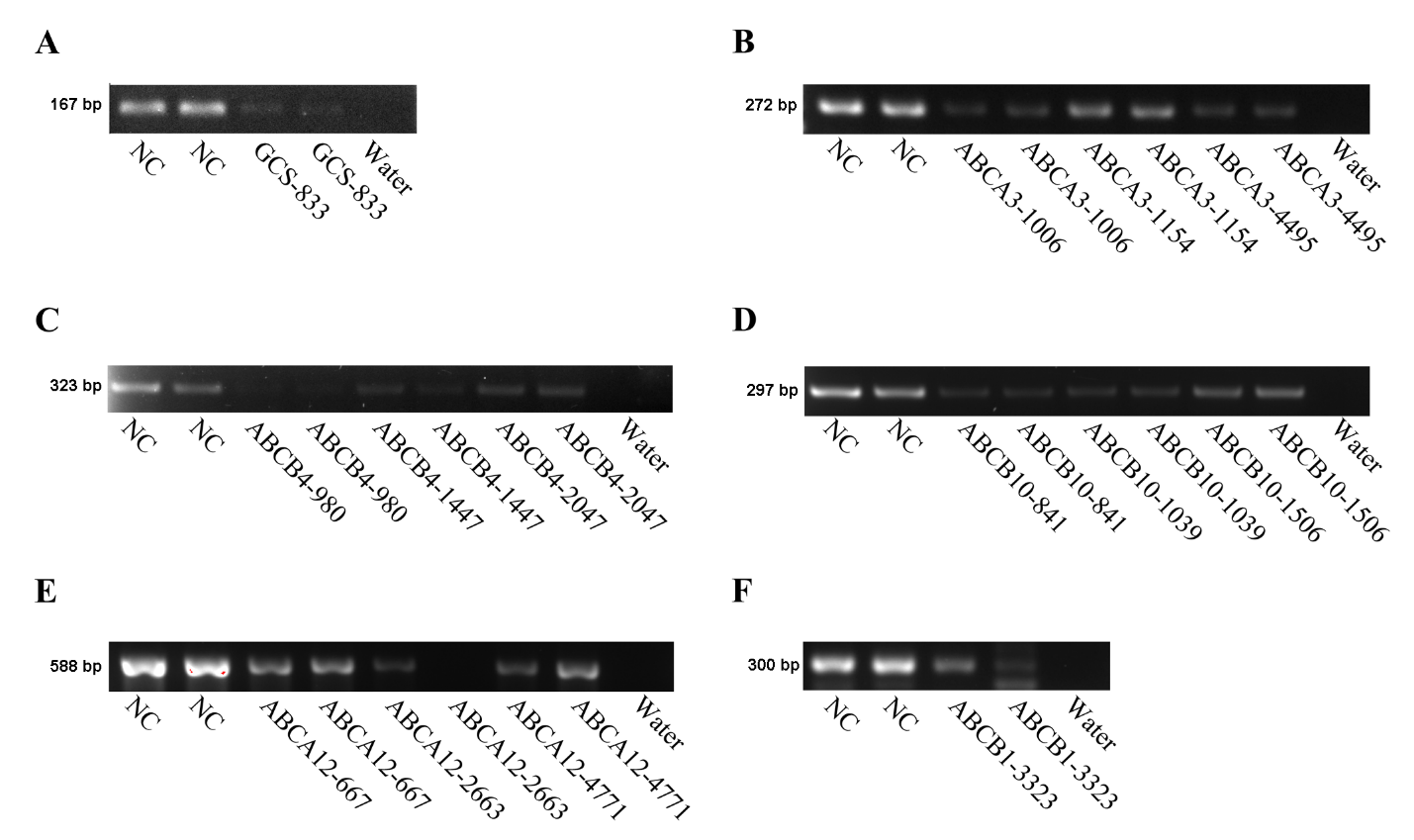
**

**Figure S2: siRNA screen in DU-145 cells.**

mRNA levels were observed by RT-PCR post 48 h transfection of DU-145 cells. Negative control siRNA scrambled sequences are indicated by NC. **A)** GCS-833 siRNA (30 nM) used in transient knockdown resulted in decreased GCS mRNA transcript. **B)** ABCA3-1006, ABCA3-1154, and ABCA3-4495 siRNA knockdowns were screened. ABCA3-1006 was the most effective in reducing ABCA3 mRNA transcript. **C)** ABCB4-980 siRNA depleted ABCB4 mRNA transcript the most compared to ABCB4-1447 and ABCB4-2046. **D)** ABCB10 RNA transcript was depleted most effectively by ABCB10-841 siRNA, compared to ABCB10-1039, and ABCB10-1506. **E)** ABCA12-2663 siRNA depleted ABCA12 mRNA transcript more than ABCA12-667and ABCA12-4771. **F)** ABCB1-3323 knockdown depleted ABCB1 mRNA transcript.

**
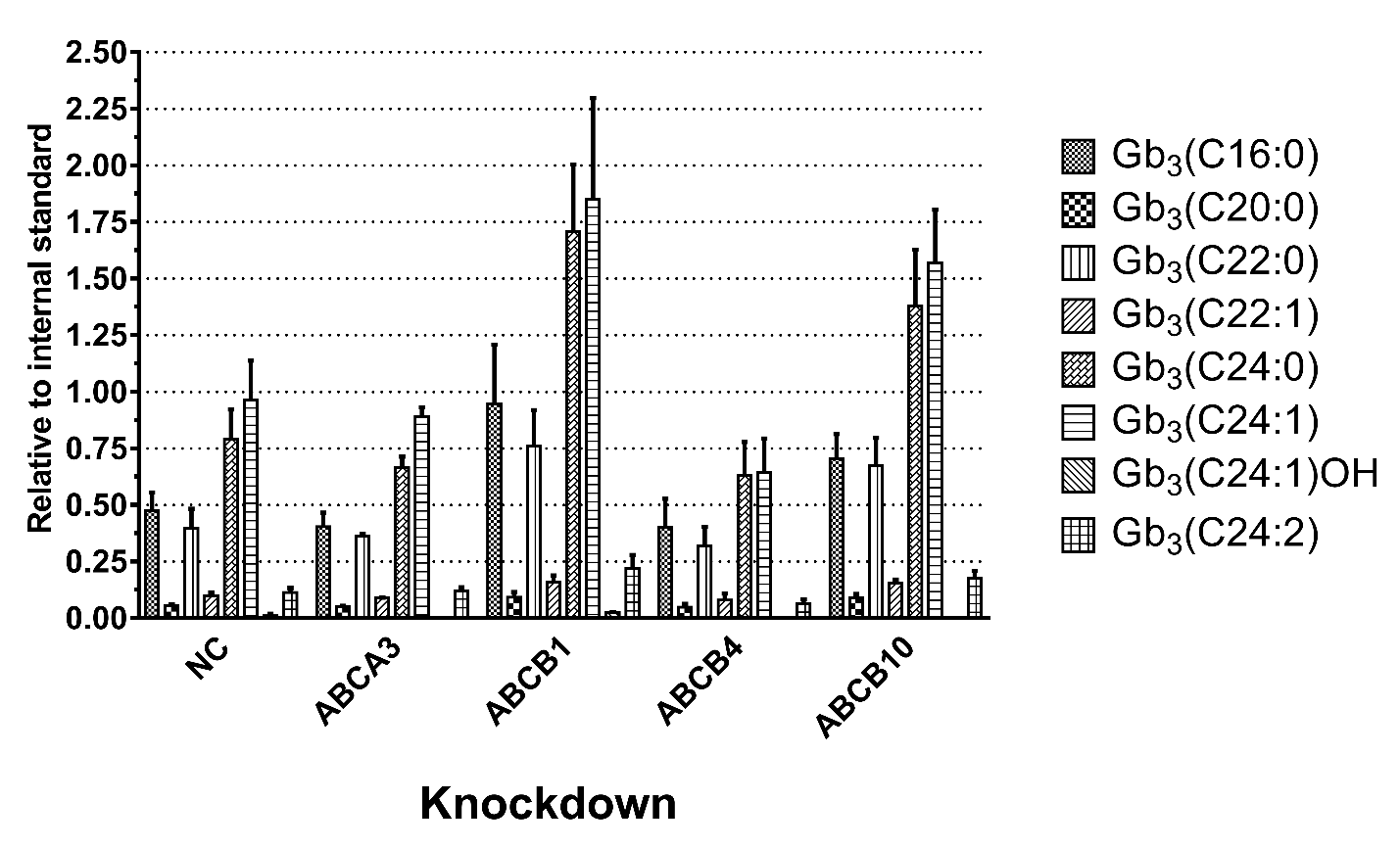
**

**Figure S3: ABC flippases have no GlcCer acyl chain preference.**

DU-145 cells were transfected with ABC transporter siRNA and Gb_3_ isoform analysis was performed by normal-phase ultraperformance liquid chromatography coupled to tandem mass spectrometry (UPLC-MS/MS) as described ([2](#_ENREF_2)). Post ABC transporter KD, no preferential Gb_3_ isoform change was observed, consistent with a lack of preference for precursor GlcCer acyl chain composition (n = 1, in duplicate). Bar graph shows mean Gb_3_ isoform levels relative to UPLC-MS/MS internal standard, standard error of mean represented as solid black lines.

References

1. Budani, M., Mylvaganam, M., Binnington, B., and Lingwood, C. (2016) Synthesis of a novel photoactivatable glucosylceramide cross-linker. *J Lipid Res* **57**, 1728-1736

2. Boutin, M., Menkovic, I., Martineau, T., Vaillancourt-Lavigueur, V., Toupin, A., and Auray-Blais, C. (2017) Separation and Analysis of Lactosylceramide, Galabiosylceramide, and Globotriaosylceramide by LC-MS/MS in Urine of Fabry Disease Patients. *Analytical chemistry* **89**, 13382-13390
